# Osteoporosis and Alveolar Bone Health in Periodontitis Niche: A Predisposing Factors-Centered Review

**DOI:** 10.3390/cells11213380

**Published:** 2022-10-26

**Authors:** Li Zhu, Chenchen Zhou, Shuo Chen, Danyuan Huang, Yukun Jiang, Yuanchen Lan, Shujuan Zou, Yuyu Li

**Affiliations:** State Key Laboratory of Oral Diseases, National Clinical Research Center for Oral Diseases, West China Hospital of Stomatology, Sichuan University, Chengdu 610041, China

**Keywords:** periodontitis, osteoporosis, hormones, metabolic disorders, lifestyle, psychological stress

## Abstract

Periodontitis is a periodontal inflammatory condition that results from disrupted periodontal host–microbe homeostasis, manifested by the destruction of tooth-supporting structures, especially inflammatory alveolar bone loss. Osteoporosis is characterized by systemic deterioration of bone mass and microarchitecture. The roles of many systemic factors have been identified in the pathogenesis of osteoporosis, including endocrine change, metabolic disorders, health-impaired behaviors and mental stress. The prevalence rate of osteoporotic fracture is in sustained elevation in the past decades. Recent studies suggest that individuals with concomitant osteoporosis are more vulnerable to periodontal impairment. Current reviews of worse periodontal status in the context of osteoporosis are limited, mainly centering on the impacts of menopausal and diabetic osteoporosis on periodontitis. Herein, this review article makes an effort to provide a comprehensive view of the relationship between osteoporosis and periodontitis, with a focus on clarifying how those risk factors in osteoporotic populations modify the alveolar bone homeostasis in the periodontitis niche.

## 1. Introduction

Bone is an organ that is in constant remodeling and turnover in the body. Bone remodeling is governed by two major types of bone cells: osteoblasts and osteoclasts. Physiological bone turnover requires efficient coordination between osteoblasts and osteoclasts. Osteoblasts derive from mesenchymal stem cells (MSCs), and the role of osteoblasts in bone metabolism differs at each maturation stage. Immature osteoblasts are capable of stimulating osteoclastogenesis, while mature osteoblasts are primarily responsible for bone organic matrix secretion and further mineralization [1]. Osteoclasts originate from hematopoietic stem cells, and the differentiation process requires the presence of two essential cytokines, receptor activator of nuclear factor-κB ligand (RANKL) and macrophage colony-stimulating factor (M-CSF) [2]. RANKL is expressed in bone marrow stromal/osteoblast precursor cells and some immune cells. RANKL binds to its receptor RANK on osteoclasts and further activates differentiation and resorptive activity of osteoclasts, during which the process of the nuclear factor (NF)-κB signaling pathway exerts a pivotal role. Besides RANKL, osteoblast lineage cells also produce a decoy receptor for RANKL, osteoprotegerin (OPG), which blocks RANKL-RANK signaling. A balanced RANKL/OPG/RANK system is of vital importance to osteoclastogensis [3]. Enhanced RANKL production and NF-κB signaling pathway activation by pro-inflammatory mediators, including IL-6, TNF-α, IL-1β, prostaglandin E2 (PGE2) and C3 are common in inflammatory diseases, including periodontitis and osteoporosis [4].

Periodontitis is a chronic infectious disease, which results from the pathological interaction between dental plaque microorganisms and the host immuno-inflammatory response [5]. An elevated proportion of some commensal bacteria in the biofilm, Gram-negative anaerobic bacteria, in particular, is closely associated with the onset and progression of periodontitis. These pathogens and their toxic products, especially lipopolysaccharide (LPS), stimulate an innate immune response. During the innate response, epithelium cells defend against bacterial invasion as a physical barrier. Locally infiltrated neutrophils could remove bacteria via phagocytosis. Meanwhile, dendritic Langerhans cells within the epithelium and mononuclear phagocyte system further represent antigens to activate lymphocytes, initiating an adaptive response. Lymphocytes immigrate to local lesions and anticipate defense against pathogens via cytotoxic T cells and the production of antibodies and pro-inflammatory cytokines. The elimination of pathogenic bacteria is often accompanied by host cellular damage during excessive immuno-inflammatory response. Therefore, both periodontal microflora and the host response, which could be modified by environmental factors, contribute to excessive inflammation and destruction of the periodontal ligament and alveolar bone in chronic periodontal infection [6]. Yu B et al. have reviewed clinical studies published between 1996 and 2000 and concluded that alveolar bone loss is in inverse correlation with systemic bone mineral density. The role of osteoporosis as a risk factor in periodontitis has been accepted [4]. Elevated inflammatory cytokines and activated NF-κB signaling drives the uncoupling of bone remodeling, promoting osteoclastic bone resorption and impairing osteogenic activity simultaneously. Therefore, an inflammation-induced bone homeostatic imbalance is considered a shared trait and mechanistic link between osteoporosis and periodontitis [2,4].

Osteoporosis is a disorder featured by reduced bone mass, deteriorated bone micro-architecture, weakened bone strength and increased fracture risk. Osteoporotic fracture rates reach up to one-third in the populations and nearly a half in postmenopausal women in Europe and the US, posing a heavy health-care burden [7]. The development of osteoporosis is associated with many systemic predisposing factors, such as endocrine changes, metabolic disorders, smoking, alcoholism and psychological stress [8]. Pharmacological agents applied in osteoporosis treatment primarily consist of anti-resorptive drugs and bone anabolic agents. Bisphosphonates and Denosumab are administrated to reduce bone catabolism. Bisphosphonates work via binding with the bone mineral component, while the latter inhibits osteoclastic bone resorption in the role of anti-RANKL monoclonal antibody. Teriparatide (PTH analog) and Romosozumab (antibody against sclerostin) dominantly act as bone anabolic agents. Anabolic agents reduce fracture risk with higher but more transient efficacy compared with anti-resorptive agents. Thus, a sequential combination would be a better choice in a clinic. Specifically, hormone replacement therapy in menopausal women cannot be overlooked. Emerging clinical and animal evidence supports that therapeutic modalities for osteoporosis are promising to help improve periodontal bone status [4,9].

**Figure 1:**
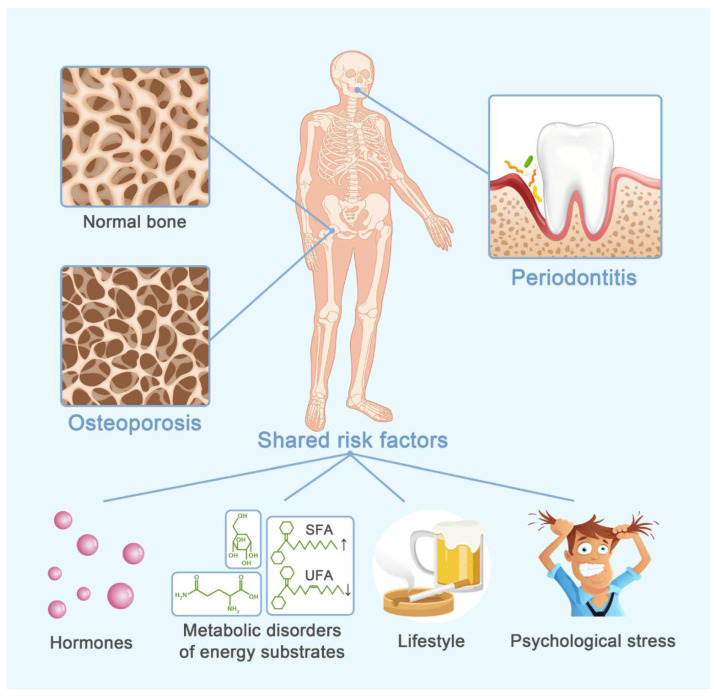
Shared predisposing factors for osteoporosis and periodontitis. Some predisposing factors might be the potential link between osteoporosis and periodontitis, including four categories: hormones, metabolic disorders of energy substrates, lifestyle and psychological stress.

It makes sense that systemic inflammation modifies periodontal cellular and molecular responses toward bacterial invasion. It is convincible that risk factors for osteoporosis affect periodontitis development [10]. Those risk factors could be divided into four categories: hormones, metabolic disorders, unhealthy lifestyle and psychological factors (Figure 1). However, periodontal niche differs from the systemic bone microenvironment, especially with the presence of periodontal pathogens. Whether those predisposing factors provoke exactly the same biological response in systemic and periodontal bone microenvironments remains unelucidated. This review focuses predominantly on periodontitis and aims to provide insight into how the systemic risk factors in osteoporotic individuals modify alveolar bone resorption in periodontitis.

We conducted an electronic search in PubMed, Scopus, Cochrane library, Web of Science without time restriction up to August 2021. The search terms are various combinations of keywords: osteoporosis, periodontitis, periodontal bone loss, endocrine, hormone, diabetes mellitus, hyperglycemia, lipid, hyperlipidemia, amino acid, lifestyle factor, smoking, cigarette, tobacco, alcohol, psychological stress and neurogenic factor. We also expanded our search into relevant literature via reference lists of reviews and identical articles. Original articles, systematic reviews and meta-analyses published after 2015 are mostly detailed and reviewed.

## 2. Hormones

Many hormones have an impact on bone, including (1) sex steroid hormones and gonadotropins; (2) calciotropic hormones, such as vitamin-D, parathyroid hormone (PTH) and calcitonin; (3) some circadian rhythm-associated hormones, such as glucocorticoids (GCs) and melatonin; (4) growth hormone (GH) and thyroid hormone (TH). Notably, complex interactions exist between other bone-regulating hormones. For instance, TH, PTH and sex steroids affect the production of GH [11,12]. GH supplementation helps restore secretion rhythm and periphery tissues sensitivity of PTH that are deteriorated under the context of GH deficiency [13] and the biological responses of target organs to sex steroids and GH stimuli are mostly interrelated [12,14].

**Figure 2:**
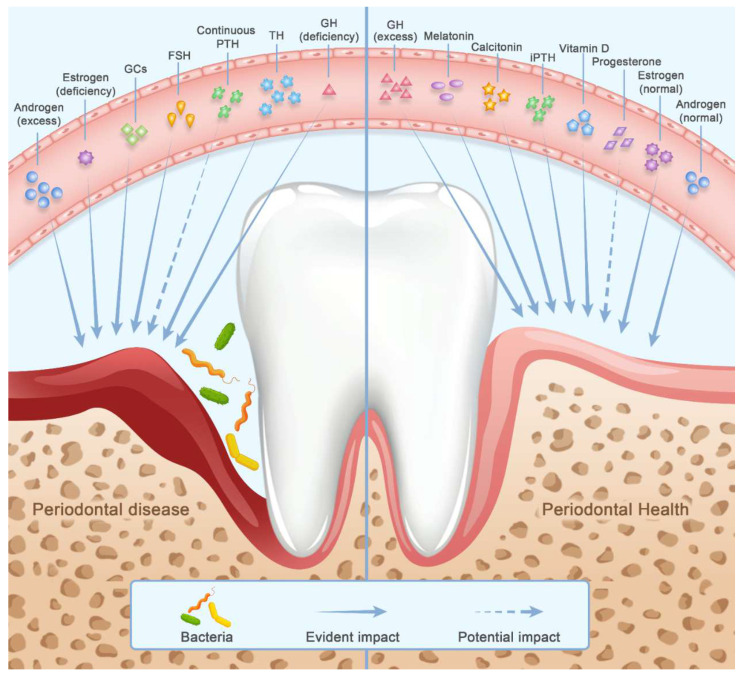
Impacts of hormones on periodontal status. There are multiple hormones that play a role in periodontal homeostasis, and their effects vary with hormone nature and circulating level. Androgen excess, estrogen deficiency, FSH, continuous PTH, GCs, TH, and GH deficiency are evident harmful factors, contributing to periodontitis. Moreover, continuous PTH is a potentially harmful factor. On the contrary, androgen and estrogen at a physiological level, progesterone, vitamin D, iPTH, calcitonin, melatonin, and GH excess are evident protective factors for periodontal health. Direct evidence for the impacts of progesterone and continuous PTH on periodontal tissues is still lacking yet. FSH follicle stimulating hormone; PTH, parathyroid hormone; GCs, glucocorticoids; TH, thyroid hormone; GH, growth hormone.

These hormones exert pleiotropic actions on skeletal and periodontal tissues in nature- and concentration-associated manner. In general, the biological effects of these hormones are mainly exerted in four ways: direct actions on bone cells and precursors via hormone receptors, indirect actions on bone cells via modifying cytokines profile, actions on periodontal ligament (PDL) cells and local biofilm, and immune-inflammatory response. In the following part, we endeavor to provide a holistic view of how these hormones independently modulate osteoporosis and periodontitis (Figure 2) (Table 1). The interaction between hormones is not the major concern of our review, but an overview of the interactive network in the future deserves to be expected.

### 2.1. Sex Steroids and Gonadotropins

Sex steroid hormones consist of estrogen, progesterone and androgen, and testosterone is the major androgen in humans. Gonadotropic hormones refer to hormones that are released from the anterior pituitary, including follicle-stimulating hormone (FSH), luteinizing hormone (LH) and human chorionic gonadotropin. Gonadotropic hormones manipulate the gonads’ growth and production of sex steroids and are modulated by sex steroids in a negative feedback manner. Aside from its significance in the development of sexual and reproductive capability, sex steroids and gonadotropins have an impact on the skeleton system as well [15]. Sex steroids protect bone health [16,17,18]. FSH induces damage to bone directly after eliminating the impact of sex steroids, and yet the influences of LH and human chorionic gonadotropin on bone remain an enigma [11].

Periodontal tissues express specific receptors for estrogen, androgen and progesterone [12]. As the target tissue of sex steroids, the periodontal condition is altered by the physiological and pathological fluctuation of these hormones. Many studies have investigated the contributions of sex steroids in gingivitis, whereas their roles in periodontitis remain unclear [12,13]. Effects of androgen, estrogen, progesterone and FSH on bone health and inflammatory periodontal bone loss will be elaborated, respectively, in the following charts.

#### 2.1.1. Androgen (Testosterone)

Androgen has been found to preserve bone mass [16]. With regard to the relationship between androgen and periodontitis, current human and animal studies reveal discordant findings. According to the data from the Third National Health and Nutrition Examination Survey (NHANES) of the United States, the relationship between testosterone level and periodontitis risk appears to be a U-shape curve. In other words, both sub-physiological levels and above-physiological levels promote periodontitis development [14]. The negative impact of increased testosterone levels on periodontal health is also supported by other investigations. However, only a limited number of studies suggest a positive correlation between low levels of testosterone and chronic periodontitis in humans [19]. Similar phenomena were observed in animal experiments. Some affirmed that both excessive high- and low-level testosterone accelerate alveolar bone loss in rats with periodontitis [20]. However, several rodent models demonstrated that depletion of testosterone weakens ligature-induced periodontal bone resorption [21,22]. To sum up, excessive testosterone is universally accepted to be detrimental to periodontal health but the effect of testosterone deficiency on periodontal tissue is still under debate.

Much effort has been made to provide insights into how androgen modulates bone health and periodontitis at the molecular level. Androgen acts with bone cells directly via specific androgen receptors and indirectly via estrogen receptor (ER)-α. Androgen enhances the expression of androgen receptors on osteoblasts, thus modulating cell proliferation, differentiation and survival. Apart from osteoblasts, cellular behaviors of osteoclasts and osteocytes are also modulated by androgen. Osteoclastogenesis is inhibited by androgen mainly through manipulating the production of RANKL and OPG from osteoblasts and their precursors. Moreover, dihydrotestosterone, a testosterone metabolite that is more potent in activating androgen receptors, can act on osteoclasts to stimulate bone resorption, and androgen suppresses osteocyte apoptosis by its aromatization into estrogen [1]. It is suggested that physiological levels of testosterone not only inhibit osteoclastogenesis in a dose-related way, but also suppress the production of TNF-α [23]. Moreover, the blockade of androgen receptors could increase bone loss and impair bone repair ability. Above-physiological level of androgen promotes osteoblast-derived osteoclastogenesis via manipulating the RANK/OPG/RANKL system. However, since inflammation impairs osteoblasts activity, the negative impact of high testosterone on alveolar bone is more significant in non-inflammatory periodontium than in periodontitis lesions [117]. Moreover, intake of exogenous anabolic androgenic steroids was found to alter the local microbiota of human periodontal tissues, increasing the proportion of some putative periodontopathic bacteria, including *Aggregatibacter actinomycetemcomitans*, *Porphyromonas gingivalis*, *Prevotella intermedia* and *Candida species* [24]. Testosterone deficiency promotes the production of pro-inflammatory mediators, enhancing inflammatory alveolar bone resorption in a rat model with experimental periodontitis [118]. However, this phenomenon was only observed in the short term after orchiectomy when IL-1α, IL-1β and IL-10 levels are increased [23,25]. Long-term depletion of testosterone ameliorates inflammation-induced production of IL-1β [21,22]. It is discovered that a reservoir of sex steroids exists in the skeletal system. After orchiectomy, sixty days are needed to eliminate residual effect of androgen [119]. In that case, androgen depletion is a protective factor for periodontal health. Taken together, androgen modifies bone cells directly and indirectly via inflammatory cytokines. Of note, it plays a role in periodontal dysbiosis as well.

#### 2.1.2. Estrogen

Estrogen deficiency is the prime etiological factor for postmenopausal osteoporosis, and it has been proven that postmenopausal osteoporosis is a predisposing factor for periodontitis progression [26]. In individuals without pre-existing periodontitis, estrogen deficiency leads to a lower proportion, higher porosity and more variable tissue mineral density of alveolar bone [120]. Intriguingly, in rat models with LPS-induced periodontitis, ovariectomy (OVX)-induced estrogen deficiency did not add to a significant reduction in alveolar bone height more than in sham groups [121].

Estrogen regulates both bone formation and bone resorption, and the latter effect occupies a dominant position [122]. Estrogen modulates bone remodeling by manipulating bone cells and cytokine profiles [123]. On one hand, estrogen-ER signaling directly curbs the differentiation and functional activity of osteoclasts [27]. Estrogen deficiency promotes cell differentiation of osteoclast progenitors by suppressing c-Jun activity. Furthermore, the deficiency reduces osteoclast apoptosis via FAS/FASL signaling and rescues the estrogen-inhibited activity of mature osteoclasts. On the other hand, estrogen promotes osteoblastic bone formation [27]. This hormone activates the Src/Shc/ERK signaling and down-regulates the c-Jun NH2-terminal protein kinase (JNK) in osteoblasts, inhibiting cell apoptosis. No agreement about the influence of estrogen on proliferation and differentiation of osteoblasts has been reached yet.

Furthermore, estrogen also manipulates the nature and level of local immune cells and mediators. Estrogen decreases the levels of pro-resorptive and pro-inflammatory cytokines. *Escherichia coli* LPS stimulated the secretion of TNF-α, IL-1β, IL-6 and RANKL in human PDL cells, and an increment of these pro-inflammatory cytokines could be inhibited by estrogen [28]. Estrogen could also slightly increase the OPG production of PDL cells [29]. ER-α knock-out mice exhibited elevated pro-inflammatory cytokines, including IL-33, TNF-α and IL-1β in periodontal tissues [30]. It is suggested that enhancement of the NOD-like receptor family, pyrin domain containing 3 (NLRP-3)/caspase-1/IL-1β signaling pathway associates with promoted bone resorption in co-morbid apical periodontitis and estrogen deficiency [31]. NLRP-3 is involved in forming the inflammasome that can be activated by antigens of some bacteria and damaged tissues. NLRP-3 activation further stimulates the caspase-1 cascade and increases the production of pro-inflammatory cytokines, including IL-18 and IL-1β [124]. In rats with installed periodontitis, although infiltration of neutrophils and T cells into periodontal lesions was reduced in OVX rats, OVX-induced estrogen deficiency promoted the capability of T cells to produce RANKL [121].

#### 2.1.3. Progesterone

The protective role of progesterone in bone health has been recognized. Optimal intake of progestin, the synthetic progesterone, is found to help prevent bone loss in premenopausal and perimenopausal women [18]. Epidemiological investigations show that elevated progesterone level is a predisposing factor for gingivitis in pregnant women, but not periodontitis in most cases. It is speculated that since periodontitis is a chronic disease, the duration of pregnancy may not be long enough to cause obvious periodontal damage [32].

The current literature demonstrates that progesterone promotes alveolar bone formation. Progesterone receptors on human PDL cells mediate action of progesterone on osteoblastic proliferation and differentiation of human PDL cells and the number of mineralized nodules was higher in progesterone-treated groups compared with the control [33]. Nevertheless, regulatory actions of progesterone on bone resorption remain less well understood. Progesterone stimulates the production of PGE2, a pro-resorption mediator [34] but progesterone also renders less secretion of IL-1β-induced matrix metalloproteinases (MMPs) secretion by gingival fibroblasts, and hence periodontal tissue degradation by MMP is suppressed [35].

#### 2.1.4. Gonadotropic Hormones

Gonadotropic hormones include FSH, LH and human chorionic gonadotropin. Recently, FSH has received much attention for its effects on bone metabolism. Both cross-sectional epidemiological investigations and rodent experiments demonstrate that FSH negatively correlates with bone health [11]. In rats with experimental periodontitis, high FSH levels secondary to OVX resulted in more alveolar bone loss compared with controls. The potentiating effects were reversed by an FSH inhibitor, triptorelin [36].

FSH receptors are expressed in osteoclast precursors and osteoclasts, but not on osteoblasts [37]. Once the FSH receptor on osteoclast precursors is stimulated by FSH, it further activates downstream osteoclastogenesis-associated changes. The FSH receptor thereafter stimulates MEK/extracellular regulated protein kinases (Erk), NF-κB and Akt (RANKL sensitive kinases) pathways, enhancing cellular transduction of RANKL signals. Moreover, FSH indirectly regulates proliferation and differentiation of osteoclast precursors via up-regulating expressions of TNF-α and RANK. The resorption of bone and apoptosis of mature osteoclasts are also under the regulation of FSH [11,38]. However, according to up to date research, it barely acts on osteoblasts. This makes sense that FSH independently impairs bone mass.

FSH could also exert an immunomodulatory effect in periodontal tissues. In rats with experimental periodontitis, high FSH levels further elevated cyclooxygenase 2 and PGE2 levels [36]. In the culture of human PDL cells, FSH up-regulated the expressions of IL-1β, IL-6 and TNF-α, as well as augmented LPS-induced production of those pro-inflammatory factors [39].

### 2.2. Calciotropic Hormones

Calcium, the main inorganic component of bone tissue, is absorbed in the intestine and kidney. Calciotropic hormones (vitamin D, PTH and calcitonin) act on bone, kidney and intestine, constructing an elaborate calcium-regulating network. They are indispensable for maintaining calcium homeostasis in the extracellular fluid. Vitamin D modulates bone remodeling and intestinal resorption of calcium. PTH acts in the kidney, intestine and bone, replenishing serum calcium during hypocalcemia. Calcitonin decreases excessive calcium load in circulation by suppressing calcium release from bone and accelerating calcium excretion from the kidney. There are complex cross-talks and tight connections among the production and biological activities of these hormones. PTH release is stimulated by calcium insufficiency and suppressed by vitamin D and the relationship between PTH and vitamin D levels is modified by age and disease. Meanwhile, PTH and high serum calcium would promote the production and release of calcitonin, while elevated calcitonin in turn decreases the serum calcium concentration and increases the production of kidney-derived calcitriol. Perturbation of this network, such as vitamin deficiency and chronic hyperparathyroidism, is possibly associated with bone disorders [40]. Therefore, we will provide an overview of how calciotropic hormones modify osteoporosis and periodontitis, in which bone loss is the shared hallmark.

#### 2.2.1. Vitamin D

Vitamin D is a class of biologically active cholesterol derivatives in the body, generally including cholecalciferol (vitamin D3), 25-hydroxycholecalciferol and 1,25-dihydroxycholecalciferol (calcitriol) [125]. Calcitriol is the active form of vitamin D3 [40]. It is acknowledged that vitamin D exerts a bone-protective effect, while vitamin D deficiency brings about a wide spectrum of bone diseases, including osteoporosis and periodontal bone loss [40,41]. Machado V et al. reviewed the relationship between vitamin D levels and susceptibility and severity of periodontitis and concluded that chronic periodontitis patients display lower 25-hydroxyvitamin D levels than their periodontally healthy peers [42]. In Norwegian and Tamil populations, the elevation of vitamin D levels is in a linear relationship with the reduction in alveolar bone loss measured on radiographs [43]. However, there are conflicting opinions on the relationship between vitamin D levels and periodontal risk in humans. Pre-existing evidence mainly derives from cross-sectional studies and clinical parameters adopted in periodontitis diagnosis among those observational investigations are inconsistent [126]. Therefore, more longitudinal studies with unified parameters are required to determine the exact relationship in populations [127]. Even so, animal studies provide supportive evidence for this association. Vitamin D insufficiency in a mice model contributed to periodontal inflammation and alveolar bone loss [128]. Calcitriol treatment attenuated periodontal inflammation and bone resorption in rat models with LPS-induced periodontitis [129].

Vitamin D displays complex regulatory effects on bone cells. On one hand, it promotes bone formation via stimulating osteoblastic differentiation, bone organic matrix synthesis and mineralization. The knockout of the gene encoding 25-hydroxyvitamin D-1α-hydroxylase in mice caused the deficient status of biologically active vitamin D, calcitriol, subsequently suppressing osteoblastic bone formation independent of mineral homeostasis (calcium, phosphorus) and age [44]. Furthermore, calcitriol was found to promote mineralization in human PDL cells in vitro [45]. On the other hand, the role of vitamin D in osteoclastic bone resorption appears confusing. In human bone tissues, vitamin D stimulates osteoclastogenesis by elevating RANKL/OPG ratio in osteoblastic lineage cells [46]. In both rat models and culture of mice cells, calcitriol was found to inhibit osteoclast formation in the role of an immunoregulatory agent, modulating T helper (Th) cells polarizing toward an anti-inflammatory and anti-osteoclastogenic subtype (Th2 and Treg cells) in the context of inflammation. Meanwhile, Th17 and Th1 cell numbers are reduced. The altered composition of the Th cell population upregulates the expression of anti-inflammatory cytokines (IL-4, IL-10), and decreases pro-inflammatory cytokines (IL-17) and RANKL/OPG ratio [47,129].

Other biological mechanisms have also been proposed to link vitamin D and periodontal status. Vitamin D might affect periodontitis due to its modulatory role in bacterial load, antibacterial system and immune-inflammatory response [127,128]. Firstly, vitamin D reduces periodontal pathogen loads to prevent the initiation of periodontitis. Calcitriol limited the intracellular growth of bacteria when administrated in the culture of human gingival epithelial cells that were infected with *P. gingivalis* [48]. Secondly, vitamin D augments host anti-microbial properties. Periodontal cells in localized periodontitis lesions express specific metabolic enzymes, turning vitamin D into calcitriol [49]. Calcitriol induces the synthesis of antimicrobial agents, including hCAP-18/LL-37 and human-β-defensin 3 antimicrobial peptide from human gingival fibroblasts and human PDL cells [50,51]. Calcitriol also reinforces the physical epithelium defense system against microbial attack via vitamin D receptors [48]. Moreover, vitamin D elevates the level of proteins vital for antibacterial autophagy in periodontitis patients [52].

Additionally, the profile of local immuno-inflammatory response is modified by vitamin D. In human gingival epithelium and periodontal ligament cells, vitamin D reduces pro-inflammatory cytokines production induced by *P. gingivalis* LPS treatment, including IL-6, IL-8, TNF-α, monocyte chemotactic protein-1 [51,53]. Sufficient vitamin D also decreases the production of matrix degradation-related enzymes, such as MMPs [127]. Vitamin D reduces the circulating cell count of cytotoxic T lymphocytes, a type of effector cell that is well-known for its dominant role in the destruction of human periodontal tissue [52]. Notably, vitamin D has also been recognized for its role in accelerating periodontal repair via manipulating immune response [127]. Studies up to date unveil part of the underlying mechanisms of the regulatory actions of vitamin D on immune-inflammatory response. As we mentioned previously, activation of the NLRP-3/caspase-1/IL-1β axis enhances infectious inflammation in an estrogen-deficient rat model. Nevertheless, this axis is inhibited by vitamin D to attenuate inflammatory response. In C57BL/6 wild-type mice with experimental periodontitis, calcitriol intervention enhances Aryl hydrocarbon receptor (AhR) signaling, which further restrains the activation of NF-κB signaling pathway in gingival epithelium. NLRP-3 inflammasome activation is further curbed due to lack of the critical trigger, phosphorylated NF-κB. The crosstalk between AhR signaling and NF-κB/NLRP-3 inflammasome pathway might be the underlying mechanism of therapeutic efficacy of vitamin D on periodontitis-related bone loss [130]. In rats with experimental diabetic periodontitis, vitamin D directly interacted with tyrosine-protein phosphatase non-receptor type 2 (PTPN2), then PTPN2 dephosphorylated CSF1R and blocked CSF1R signaling. Since CSF1R signaling is of crucial importance to osteoclastogenesis, it is plausible that vitamin D-induced attenuation of alveolar bone resorption should be partly accredited to the PTPN2/CSF1R pathway [131]. Although vitamin D has been proven to affect periodontal pathology, there is only weak evidence for the therapeutic promise of vitamin D supplementation in periodontitis. This may be accredited to inflammation-induced down-expression of vitamin D receptors in PDL cells [132].

#### 2.2.2. Parathyroid Hormone

PTH, an 84-amino acid endogenous hormone (PTH 1-84), is responsible for adjusting extracellular calcium and phosphate. The influence of PTH on bone metabolism differs with administration mode, in other words, intermittent PTH (iPTH) or continuous PTH (such as chronic hyperparathyroidism). Continuous PTH causes hyperactive bone resorption and mild bone formation, exerting a catabolic effect on bone metabolism. Conversely, the anabolic effect of iPTH on bone metabolism has been verified. Teriparatide, an active fragment of PTH (PTH 1-34), has been approved for osteoporosis therapy in intermittent administration mode by the Food and Drug Administration [54]. Moreover, iPTH is able to prevent alveolar bone loss in experimental periodontitis and enhance the periodontal reparative capability of rodents, including alveolar bone regeneration [55]. The promise of iPTH in periodontitis treatment is also supported by human clinical trials [56].

The mechanisms of how PTH modulates cellular behaviors of bone-forming cells are comprehensive. PTH type 1 receptor (PTH1R) is expressed in the osteoblast-lineage cells but not on osteoclasts. Hence, PTH indirectly promotes osteoclastogenesis by increasing the local production of RANKL and M-CSF [65]. In fact, osteocytes are found to occupy a pivotal position in response to PTH. PTH induces osteocytes to down-regulate the expression of SOST, which encodes a bone formation inhibitor, sclerostin [57]. Enhanced RANKL expression in osteocytes stimulated by PTH is the key driver for osteoclast formation [58]. Furthermore, PTH-triggered “osteocytic osteolysis” enables rapid serum calcium replenishment in hypocalcemia [59]. The most significant disparity between the actions of iPTH and continuous PTH on bone lies in osteoblastic bone formation. iPTH modulates the proliferation and differentiation of osteoblast precursors and enhances the survival and functional activity of osteoblasts. Multiple molecules are involved in the anabolic actions of iPTH on osteoblast lineages, especially in iPTH-induced inhibition of apoptosis of osteoblast lineages [54,60,61]. As to the reason why continuous PTH drives different skeletal responses with iPTH, there are two possibilities [54]. One is that continuous PTH decreases the cell number of osteoblasts by infringing the stability of Runx2, as well as not mitigating osteoblast apoptosis. The other hypothesis focuses on osteoclast/osteoblast interaction. During osteoclastic resorption, the breakdown of bone organic matrix releases growth factors, such as insulin-like growth factor (IGF)-1 and transforming growth factor (TGF)-β1. These growth factors facilitate the immigration and differentiation of osteoblast progenitors. However, continuous PTH is prone to depleting the growth factors reservoir. It is worth noting that this phenomenon also occurs during iPTH administration after long-term treatment, manifested by the decline of bone anabolic actions over time.

PTH1R is also expressed in PDL cells, displaying higher density, lower binding affinity and different downstream signaling compared with osteoblasts [133]. It has been found that the responses of human PDL cells to PTH stimuli depend on the maturation state of cells and PTH administration mode. With iPTH treatment, the number of cells decreased in pre-confluent PDL cells but increased in confluent PDL cells. iPTH also promoted osteoblastic differentiation (measured by ALP activity and OCN) and expression of OPG in pre-confluent PDL cell while completely opposite effects were observed in confluent PDL cells. However, iPTH did not bring obvious change to the expression of RANKL in both cultures [62]. In other words, iPTH stimulates osteoblastogenesis and indirectly suppresses osteoclastogenesis via a reduced RANKL/OPG ratio in the culture of pre-confluent PDL cells. On the contrary, iPTH exerts opposite effects on more confluent PDL cells. With regard to the influence of continuous PTH on PDL cells, available data indicate that cell proliferation is not affected by continuous PTH regardless of maturation state [63]. In addition, continuous PTH stimulates osteogenic differentiation of human PDL cells in a short time after application, evidenced by elevated expression of ALP [134]. Molecular mechanisms underlying the impact of PTH (iPTH and continuous PTH) on PDL cells have been explored. PKA- and PKC-dependent pathways participate in the regulation of PTH on the proliferation, differentiation and survival of human PDL cells. Both pathways could manipulate ERK 1/2 component, an essential component during cell proliferation. In response to iPTH stimuli, activated PKC along with ERK1/2 stimulates the proliferation of PDL cells, while the anti-proliferation action of PKA is achieved partly by inhibiting ERK1/2. PKC and PKA both can inhibit cell apoptosis, and the latter is more potent [135]. Additionally, iPTH (1-34) modulates the expression of OPG in PDL cells via PKA-dependent pathway [136]. Therefore, iPTH modifies activity of PKA and PKC signaling pathway, causing complex biological responses of PDL cells in different mature stages. The PKC-dependent pathway is also involved in continuous PTH-induced transitorily osteogenic differentiation of human PDL cells by upregulating the expression of ALP [134]. Those findings indicate that PTH treatment affect both proliferation and osteogenic differentiation of PDL cells in an administration mode- and cell maturation-dependent manner.

The roles of some inflammatory mediators in PTH actions have been noticed. iPTH decreases local levels of IL-6, MMP-2 and MMP-9 in Wistar rats with experimental periodontitis [64]. Moreover, PTH stimuli on PDL fibroblasts caused a transient decrease and then elevation of PGE2 production, indicating that PTH regulates inflammation and destruction of periodontal tissue in a time-specific pattern [66]. It is proven that the soluble IL-6 receptor is critical in mediating the biological effects of PTH on bone. PTH activates soluble IL-6 receptor/gp130/STAT3 signaling pathway in myeloid lineages and thus increases the secretion of TGF-β, contributing to the bone anabolic effect of PTH [137].

#### 2.2.3. Calcitonin

Calcitonin is an ancient hormone in the biological evolution process, and primarily restrains the magnitude of hypercalcemia. However, different from the well-accepted regulatory actions of vitamin D and PTH on bone metabolism, current knowledge regarding the biological effects of calcitonin on bone are limited except for bone mass maintenance during lactation [40]. An overview of the exact role of calcitonin in bone metabolism is still required yet.

The role of calcitonin in bone health is intriguing. Investigations on human beings reveal the promise of calcitonin in preventing the decline of bone mineral density and lowering osteoporotic fracture risk [67]. Moreover, calcitonin receptor gene polymorphism correlates with osteoporosis [68]. In rats with calcitonin gene knockout, bone mass and quality were improved, while the activity of bone resorption is similar to wide-type controls when these rats are younger than the age of 12 months. After that time, bone resorption was increased significantly [69]. These in vivo data demonstrate that calcitonin inhibits both bone formation and bone resorption. It is attractive to find that calcitonin is likely to modify periodontitis and related tissue destruction. Calcitonin concentration in gingival crevicular fluid (GCF) of periodontitis patients are higher than controls, correlating with periodontal clinical parameters [70]. Moreover, calcitonin receptor gene polymorphism tends to correlate with periodontitis risk [68]. In Wistar rat model with experimental periodontitis, both systemic and local administration of calcitonin mitigated alveolar bone loss [73,74].

The osteoclast is the major cell component that responds to calcitonin stimuli during bone metabolism. Calcitonin suppresses migration and differentiation of osteoclast precursors. Upon binding with the calcitonin receptor on osteoclasts, calcitonin activates downstream cyclic adenosine phosphate signaling in mice and phospholipase C pathway in humans, inhibiting the formation of sealing zone and the synthesis of resorption-associated enzymes [71,72]. Notably, the inhibitory effects of calcitonin on osteoclasts are transient owing to decreased expressions of calcitonin receptors on osteoclasts by continuous calcitonin stimulus [71]. Therefore, intermittent administration of calcitonin may be feasible to maximally exert its anti-resorption effect. A similar inhibitive effect on osteoclasts of calcitonin has also been observed in periodontitis [73,74].

Calcitonin impinges osteoblastic bone formation via two major pathways. One is that calcitonin promotes cell survival and upregulates the expression of sclerostin in osteocytes, while sclerostin acts as a potent inhibitor of bone formation. The other mechanistic pathway is that calcitonin acts on the hypothalamus and thus manipulates the neuroendocrine signaling network that is involved in bone turnover [73]. Nevertheless, calcitonin displays anabolic actions on periodontal bone. Over-expression of calcitonin in human PDL fibroblasts induces the expression of the bone morphogenetic protein (BMP), ALP and OCN, as well as the production of the bone organic matrix (mostly collagen). Mechanistically, TGF-β1 and BMP signaling pathway, respectively, mediates the differentiation and matrix synthesis processes of human PDL fibroblasts in response to calcitonin [70].

### 2.3. Circadian Physiology-Associated Hormones

The circadian clock system is composed of the suprachiasmatic nucleus in the brain (central pacemaker) and circadian machinery in the peripheral tissues (molecular oscillators). The hypothalamic–pituitary–adrenal (HPA) axis and sympathetic nervous system (SNS) are responsible for the signal transfer from the suprachiasmatic nucleus to peripheral tissues, manipulating the coordination of various physiological processes. Emerging evidence indicates that bone metabolism is associated with circadian rhythm [74]. Both the etiology and prognosis of osteoporosis are modulated by the circadian system. The influence of the circadian system on bone homeostasis is primarily mediated by several pivotal hormones, including GCs and melatonin.

#### 2.3.1. Glucocorticoids

GCs contain endogenous hormones (cortisol in primates, corticosterone in rodents) and synthetic compounds. The physiologically diurnal fluctuation of GCs contributes to preserving bone health [74]. Excessive circulating GCs are reported to correlate with osteoporosis and fracture risk [75]. Circadian dysrhythmia, chronic physiological stress and long-term GCs therapy could disturb the secretion of GCs primarily via the HPA axis [74,76]. Whether GC-induced osteoporotic change exists in periodontal bone has not been clarified yet. Some studies support that GCs administration induces the osteoporotic change of alveolar bone [77]. However, another study proposed that long-term GC treatment indeed brought about an osteoporotic change in the mandibular bone but not in the bone which surrounds and supports the tooth [138]. It is intriguing to find out how GCs affect periodontal tissues in a periodontitis niche. Elevated cortisol induced by psychological stress was shown to correlate with susceptibility and severity of periodontitis in the human body [78]. Data derived from rodent experiments corroborate clinical evidence. In rats with either genetic or experimental hyper-reactivity of the HPA axis, high circulating GCs potentiate ligature-induced periodontal destruction when compared to rats with an HPA axis of low responsiveness [139]. Before ligature, administration of GCs receptor antagonist, RU 486, could reverse the adverse effects of the highly activated HPA axis on periodontal tissues [140]. Additionally, Breivik et al. revealed that stressors applied to activate the HPA system in neonatal rats could result in persistent suppression of the HPA axis reactivity due to the down-expression of GR in the hypothalamus in adulthood of those rats. Low responsiveness of the HPA system to danger signals further leads to lower response to LPS stimuli and decreased predisposition to periodontitis [141]. Collectively, the high-responsiveness of the HPA axis increases periodontitis risk and tissue destruction, while the low-responsiveness of the HPA axis displays opposite effects.

GCs disturb bone homeostasis via direct actions on bone cells. Excessive GCs suppress bone formation via restraining differentiation and survival of osteoblasts. Mechanistic studies reveal that excessive GCs up-regulate the expression of sclerostin, causing enhancement of peroxisome proliferator-activated receptor gamma receptor 2 (PPARγ2) signaling and inhibition of the Wnt/β-catenin pathway. Moreover, excessive GCs promote bone resorption. It modulates RANKL/OPG/RANK system, and thus increases the cell number and function of osteoclasts. Since critical cytokines in GC-induced osteoclastogenesis are mainly osteoblast-derived, it is plausible that aggravated bone resorption occurs only transiently in the early stage under the context of excessive GCs [75].

Furthermore, researchers reported that GCs exhibit dual regulatory roles on inflammation, in other words, concurrent pro-inflammatory and anti-inflammatory actions. Those two types of actions are both parts of the strategy to maintain host homeostasis. The overall inflammatory profile in response to GCs stimulus varies with lineages, differentiation stages and metabolic states of cells. The inflammatory response is further modified by administration timing and dosage of GCs. The discrepancy in administration order (prior to or after the initiation of inflammation) and treatment duration also account for different inflammatory profiles. When administrated with low concentration and/or prior to the onset of inflammation, GCs generally induce a pro-inflammatory response. Conversely, when with pre-existing immune stimuli and/or high doses, GCs administration displays anti-inflammatory actions [76,79]. Hence, under conditions of pre-existing periodontitis, GCs display anti-inflammatory properties. However, a high level of GCs brings about a compensatory decline of GR expression in periodontal tissues, concealing the anti-inflammatory effects of GCs. In rat models, when experimental periodontitis is induced prior to chronic physiological stress, the number of plaques, as well as levels of pro-inflammatory cytokines and alveolar bone loss in the experimental group were all higher than controls with experimental periodontitis only. Although circulating corticosterone is elevated, the GR-α signaling pathway was found to be restrained owing to the down-expression of GR-α. The phenomenon that psychological stress aggravates alveolar bone loss is only observed in the context of pre-existing periodontitis, but not in clinically healthy periodontal tissues. It is speculated that GR-α mediates the anti-inflammatory effects of GCs, and inflammatory niche possibly renders periodontal tissues more sensitive to silencing of GR-α signaling [80]. Clinical trials also demonstrate that elevated circulating cortisol owing to social adversity-induced psychological stress promoted the activity of neutrophils and load of local bacteria (e.g., *Bacteroides forsythus*, *P. gingivalis*) in periodontal tissues, accounting for accelerated tissue destruction and alveolar bone loss in periodontitis [142].

#### 2.3.2. Melatonin

Melatonin is an important hormone primarily secreted by the pineal gland. The level of melatonin fluctuates with the environmental light/dark cycle, peaking in the dark environment and declining with light. Melatonin is responsible for conveying information pertaining to circadian rhythm to peripheral organs. Serum melatonin enters into the oral cavity via saliva and mucous epithelium, protecting from some oral diseases, such as periodontitis [81]. In the past decades, it has been recognized that melatonin maintains the metabolic homeostasis of bone and protects against various bone metabolic disorders, including osteoporosis and periodontitis [82]. Melatonin insufficiency contributes to aging, menopause and diabetes-associated osteoporosis, and supplementation of which helps improve bone status [82,83]. However, the results of studies focusing on the relationship between periodontitis and melatonin are conflicting [84]. Clinical studies indicate that oral melatonin supplementation arrests periodontitis progression and alveolar bone destruction, evidenced by improved clinical parameters and reduced salivary RANKL/OPG ratio, regardless of the application of nonsurgical periodontal therapy [85]. Whereas Konečná B et al. reported that melatonin application displays no impact on periodontitis, measured by clinical parameters and oxidative stress markers in saliva, in both animal models and humans [143].

Melatonin is a pleiotropic hormone. It could directly act with bone cells. Melatonin promotes the differentiation and functional activity of osteoblasts [81] and inhibits osteoclast differentiation by preventing RANKL-RANK interaction and increasing osteoblast-derived OPG [86]. Except direct regulation of bone remodeling, melatonin could also act as antimicrobial, immunomodulatory, antioxidative and pro-angiogenic agents to modify the pathophysiological process of periodontitis [87]. Melatonin inhibits the growth of many pathogens, such as *P. gingivalis*, and suppresses biofilm formation [88]. It also modulates the process of angiogenesis via vascular endothelial growth factor (VEGF), a potent pro-angiogenic cytokine, probably accelerating bone healing in rats [89]. The majority of studies suggest that melatonin administration attenuates inflammatory response and oxidative stress in periodontitis, assisting in suppressing the onset and progression of periodontitis and facilitating the repair of periodontal tissue [144]. Melatonin decreases the production of pro-inflammatory mediators via TLR4/myeloid differentiation factor 88 (TLR4/MyD88) pathway and further rebalances RANKL/OPG/RANK system, ameliorating inflammatory alveolar bone resorption in rats with experimental periodontitis [90]. In addition, the migration of immune cells, such as Th1 cells, is also modified by melatonin [91]. Apart from immuno-inflammatory responsiveness, oxidative homeostatic imbalance also affects periodontal bone metabolism by launching an oxidative attack on bone cells [87]. Melatonin can also neutralize oxidative stress induced by radiotherapy and hyperglycemia, subsequently maintaining bone homeostasis [92,93]. Moreover, melatonin possesses the capability of regulating glucose homeostasis and circadian rhythms, and thus ameliorates periodontitis with concomitant diabetes mellitus or psychiatric disorders [81].

### 2.4. Thyroid Hormone

TH is synthesized by the thyroid gland and released into the bloodstream. TH consists of thyroxine (T4) and triiodothyronine (T3) in humans, and the latter acts as the biologically active form in regulating skeletal development and bone metabolism. Notably, the overall effect of TH on bone homeostasis is concentration-dependent. Current data demonstrate that the physiological concentration of T3 exhibits anabolic actions on bone, while under conditions of either hyperthyroidism or hypothyroidism, bone health is impaired [94]. Correlative interaction between TH disorders and periodontitis has also been established and hypothyroidism is proven to aggravate alveolar bone loss in periodontitis patients [104]. Intensifying the effect of TH deficiency on periodontitis severity was affirmed by an investigation in 538 patients [105] and this correlation was also supported by the latest scoping review [106]. A cross-sectional study proposed conflicting results, indicating that TH supplementation in patients with hypothyroidism failed to take effect in terms of improving periodontal status regardless of treatment dosage and duration [145]. However, this finding needs further verification considering many inherent limitations of this study. Aside from hypothyroidism, hyperthyroidism has also been recognized for its impact on periodontitis. It was reported that thyroid disorders are associated with an increased prevalence rate of periodontal inflammation and tissue destruction, measured by clinical parameters and inflammatory markers. This correlative relationship is more evident in hyperthyroidism populations than in those with hypothyroidism [95,96]. The results of animal studies corroborate clinical findings. In rats with experimental periodontitis, hyperthyroidism is more potent in affecting periodontal bone metabolism than hypothyroidism, presented by calcium-phosphorus metabolism, OCN concentration and phosphatases activity [97].

TH nuclear receptors (TRs) are expressed in osteoblastic lineages and osteoclasts, mediating regulatory actions of TH on the skeletal system. TRs contain four isoforms, TRα1, TRα2, TRβ1 and TRβ2 [98]. It has been found that TRα is the predominant receptor under the context of sustained abnormal levels of TH, while the transient deviation of TH concentration affects bone homeostasis mainly via TRβ in mice [99]. T3 exhibits both anabolic and catabolic actions on bone at cellular and molecular levels. T3 stimulates the proliferation and differentiation of osteoblast progenitor cells and enhances the synthesis of the organic matrix as well. T3 also promotes osteoclastic bone resorption, which could partly be accredited to augmented actions of some inflammatory cytokines, including IL-1, IL-6 and IL-8 [98]. In addition to directly manipulating bone effector cells, other indirect pathways, such as GH/IGF-1 pathway, are highly likely to contribute to the actions of TH on bone [99].

Aside from effects on cellular behaviors of osteoblasts and osteoclasts, TH further modifies oxidative homeostasis, immuno-inflammatory responses and local microbiome in periodontitis tissue. Thyroid dysfunctions tend to intensify oxidative stress in periodontitis, enhancing the amplitude of both lipids peroxide oxidation and protein oxidative modifications [100,101]. Compared to controls with periodontitis only, rats with comorbidity of experimental periodontitis and thyroid disorders were found to have more neutrophils with decreased transmembrane potential, which further triggered cellular apoptosis of neutrophils by overproducing reactive oxygen species (ROS) and destructing the mitochondrial inner membrane. Since neutrophils are critical components of nonspecific immune response in response to bacterial invasion, immoderate death of neutrophils and ROS release partly account for aggravated periodontitis-related tissue destruction by thyroid dysfunction [102]. Consistent with clinical studies that reveal stronger effects of hyperthyroidism on periodontitis, augmentations of oxidative stress and neutrophil apoptosis are both more pronounced in hyperthyroidism than in hypothyroidism. Two pivotal inflammatory mediators in thyroid disorders, IL-6 and TNF-α, also play a role in inflammation and tissue destruction in periodontitis [102]. In addition, thyroid disorders aggravate oral dysbiosis in rats with experimental periodontitis, increasing the diversity and quantity of periodontal pathogen species [103]. Oral dysbacteriosis induces an immuno-inflammatory response, resulting in overloads of neutrophils, ROS and degeneration-related enzymes in periodontal lesions.

### 2.5. Growth Hormone

GH is a pituitary hormone that exerts pleiotropic effects on bone biology. Dysregulated GH is the major etiology of many diseases. Excessive GH results in acromegaly in adults, while deficient GH causes isolated GH deficiency. GH-associated disorders, including acromegaly, are suggested to correlate with osteoporosis [107,108,115]. Disputes exist pertaining to the therapeutic effect of GH on age-related osteoporosis, which may be related to differential IGF-1 levels at baseline among studies. Osteoporotic patients with low circulating IGF-1 levels may be more sensitive to GH therapy than those with a high concentration of IGF-1 [115]. In aged populations with osteoporosis, GH therapy reduces fracture risk along with a non-significant change in bone mineral density. It is speculated that other parameters of bone health, such as bone quality, that cannot be measured by densitometric endpoints might also benefit from GH therapy [108]. Similarly, GH replacement therapy could relieve adverse manifestations of the skeletal system and decrease osteoporotic fracture risk in hypopituitary adults [116]. Nevertheless, the influence of abnormal GH levels on periodontal tissues are not exactly the same. Epidemiological data support the increased risk for individuals with isolated GH deficiency to suffer from periodontitis and have greater probing depth and attachment loss than their healthy peers [146]. Adults with isolated GH deficiency. Quite opposite to GH deficiency, clinical studies show that acromegalic populations suffer from periodontitis with a lower prevalence rate and less severity [147].

GH modulates bone homeostasis via both direct and indirect approaches. On one hand, GH regulates bone metabolism directly via the specific receptor. On the other hand, IGF-1 derived from the liver and bone are both elevated in response to GH stimulus, mediating anabolic actions of GH on bone in both endocrine and paracrine manner [115]. It is generally referred to as GH/IGF-1 axis. Overall, GH and IGF-1 are both anabolic factors for bone metabolism. GH/IGF-1 axis promotes the proliferation, differentiation and secretion function of osteoblastic lineages and IGF-1 closely associates with the mineralization of bone matrix. Additionally, GH/IGF-1 axis also facilitates the formation, maturation and bone resorption activity of osteoclasts [109]. There is a slight distinction between GH and IGF-1 with regard to regulatory actions on osteoclastogenesis. IGF-1 stimulates osteoclasts differentiation by increased production of RANKL, which is blunted by GH slightly via direct induction of OPG synthesis [115]. Furthermore, GH/IGF-1 axis helps coordinate bone remodeling via proper osteoblast–osteoclast coupling, and this action depends on IGF-1-induced expression of ephrin B2 (a membrane-bound ligand) and EphB4 (its receptor) [110]. On the whole, GH/IGF-1 axis accelerates bone formation and bone resorption simultaneously and tips the balance towards new bone formation under physiological conditions. GH excess increases bone turnover, while GH deficiency lowers the bone turnover rate. Both conditions indeed impair bone health.

The expression of both the GH receptor and IGF-1 receptor is detected in periodontal tissues [148]. Investigations in past decades have offered some other valuable clues pertaining to the influence of GH on periodontal bone metabolism. Increased BMP-2, an important mediator in periodontal regeneration, in the GCF is implicated to have relevance with mitigated chronic periodontitis under the context of excessive GH/IGF-1 in acromegaly patients [111]. In parallel to clinical findings, in vitro experiment reveals that GH administration enhances the expression of osteogenic genes, including BMP and ALP, in both human alveolar bone-derived osteoblastic lineages and human PDL cells. The regulatory effects of GH on those cells are cell nature-, mature state- and time-specific. Long-term GH application boosts mineralization but not cell proliferation in the culture of alveolar bone-derived osteoblastic lineages, shown as the elevated formation of mineralized nodes compared to the culture of PDL cells [112]. According to the result of this study and the mitogenic effect of GH on human osteoblasts precursors, it is speculated that bone anabolism induced by GH is composed of sequential actions. In other words, it stimulates the proliferative activity of osteoblast progenitors and then enhances differentiation and functions when they reach the post-proliferative stage.

Under the condition of GH-related disorders, periodontal alteration is partly ascribed to the local immunological profile. High GH in acromegalics is found to arrest inflammatory periodontal destruction via modifying inflammatory response and collagen metabolism. Levels of IL-1, IL-10 and carboxyterminal telopeptide of type I in acromegalics are lower than in patients with periodontitis only [113]. Immuno-inflammatory mediators, including C-reactive protein, MMP-8 and IL-8, are elevated in periodontal pockets of individuals with isolated GH deficiency [114].

## 3. Metabolic Disorders of Energy Substrates

Bone remodeling, a dynamic process, occurs in numerous niches that are constituted of osteoblasts, osteoclasts and osteocytes. Osteoblastic bone formation and osteoclastic bone resorption are both highly energy-demanded processes. Glucose, fatty acids (FAs) and amino acids (AAs) are three major fuel substrates utilized by bone cells during remodeling. Special attention has been paid to the underlying mechanisms of bone metabolic disorders from the perspective of energy metabolism in the past decades [149].

It is accepted that diseases of substrate metabolism, such as diabetes mellitus, obesity and anorexia nervosa, contribute to the onset and development of osteoporosis [150]. Systemically disturbed energy homeostasis would interfere with fuel utilization of bone cells (bioenergetic reprogramming) and thus affect their differentiation and function, which eventually disturbs the elaborate balance between bone formation and bone resorption [149]. Herein, we will introduce the bioenergetic of bone cells first. Afterwards, contributions of dysregulated metabolism of glucose/FAs/AAs to bone metabolic disorders (osteoporosis and periodontitis) will be elaborated, respectively, with a specific mention of bioenergetic reprogramming of bone cells under these pathological conditions.

### 3.1. Bioenergetics of Bone Cells

Glucose, FAs and AAs can be utilized by bone cells to generate adenosine 5′-triphosphate (ATP) in support of various cellular physiological activities. All three nutrients could be metabolized via oxidative phosphorylation in mitochondrial respiration. Furthermore, glucose could yield energy via glycolysis in the cytoplasm [149]. Apart from energy output, metabolic pathways including oxidative phosphorylation and glycolysis also affect cellular homeostasis and functions by generating various metabolites.

**Figure 3:**
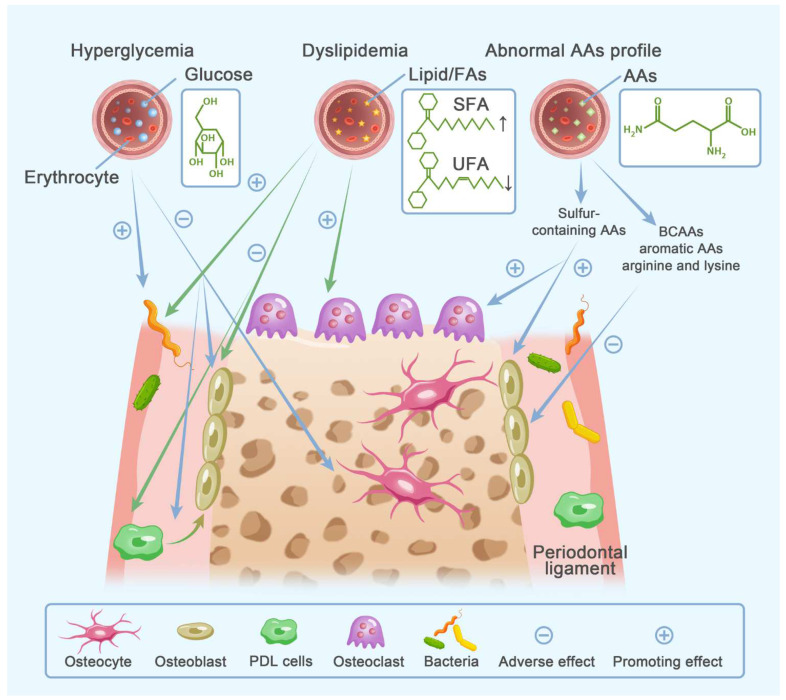
Impacts of metabolic disorders of energy substrates on periodontal status. Hyperglycemia, dyslipidemia and abnormal AAs profile are common manifestations of disordered metabolism of energy substrates. They display comprehensive actions on periodontal components, including PDL cells, osteoblasts, osteoclasts, osteocytes and bacteria. The symbol “+” indicates promoting effect, and symbol “−” indicates adverse effect. FAs, fatty acids; SFA, saturated fatty acid; UFA, unsaturated fatty acids; AAs, amino acids; PDL, periodontal ligament.

Bone formation and bone resorption both require a huge amount of energy. The bioenergetic profiles of bone cells are cell type- and stage-specific. Glucose is the preferred substrate for all bone cells. FAs and AAs modulate bone remodeling in many ways, including acting as alternative fuel substrates. Since the metabolic plasticity of bone cells is closely connected with cell differentiated phenotype, we speculate that reprogrammed bioenergetics of bone cells might link fuel metabolic disorders with pathological bone metabolism. Moreover, energy substrates could also be involved in bone metabolism in the role of extracellular signaling molecules. Hence, current evidence pertaining to how clinical disorders of substrate metabolism connect with bone metabolic disorders will be reviewed in the following parts (Figure 3) (Table 2).

### 3.2. Clinical Relevance

#### 3.2.1. Glucose Metabolism

Dysregulated glucose metabolism could bring about various clinical disorders, represented by diabetes mellitus. Diabetes mellitus possesses several hallmarks: hyperglycemia, excessive ROS, and advanced glycation end-products (AGEs). AGEs are generated after glucose links with protein or lipid. Sustained hyperglycemia along with oxidative stress, leads to excessive production of AGEs [237]. DM is divided into two types, type 1 DM and type 2 DM. Type 1 DM is caused by insulin insufficiency. As an auto-immune disease, auto-reactive CD8^+^ T cells are proven to associate with the destruction of pancreatic beta cells in type 1 DM [238]. Type 2 DM is associated with insulin resistance, and blood insulin is normal and even high in these populations. Abnormity of both innate and adaptive immune systems, including the altered cellular proliferation of T cells and macrophages, as well as dysfunction of B cells and NK cells, have been observed in type 2 DM [239]. Both types of DM correlate with osteoporosis. Type 1 DM patients display reduced bone mineral density. Despite type 2 DM presenting even higher bone mineral density, increased skeletal fragility still contributes to fracture risk in this population [151]. Although diabetes mellitus disturbs bone metabolism and diabetic patients are susceptible to osteoporotic fracture, direct evidence supporting that abnormal glucose level is a risk factor for systemic bone mineral density decline is limited [240]. Bone fragility means not only reduced bone mass but also deteriorated bone quality [237]. Under conditions of dysregulated glucose metabolism, AGE accumulation in extracellular bone matrix causes excessive AGE-collagen cross links, which subsequently elevate collagen brittleness and impair the strength and flexibility of bone [152]. Periodontitis-associated bone loss has been accepted as another common diabetic complication, in which inflammatory tissue destruction is more severe than that in non-diabetic peers. Impaired glycemic tolerance acts as a prime determinant of elevated incidence and severity of periodontitis in diabetic, pre-diabetic and non-diabetic individuals when compared to controls with glycemic homeostasis [153].

Considering that hyperglycemia acts as a primer etiology in diabetic osteoporosis, it is intriguing to find out whether pharmacological agents for DM control help improve bone health. Mohsin S, et al. have reviewed the therapies for diabetic osteoporosis treatment. As the most common treatment choice for DM, insulin therapy and glycemic control are reviewed as anti-inflammatories. However, the impact of insulin treatment on osteoporosis is inconclusive [241]. Differences pertaining to the effect on bone exist among other anti-DM agents. Specifically, how immunological profiles work in those processes is unclear. Glucagon-like peptide (GLP)-1 agonists and metformin are more evident in protecting bone health. Nevertheless, actions of other drugs, including alpha-glucosidase inhibitors, sulfonylureas, meglitinides and DPP4 inhibitors, on osteoporosis are controversial and are inclined to a neutral effect. Thiazolidinediones and sodium-glucose co-transporter 2 (SGLT2) inhibitors are even reported to exert adverse effects on bone [151].

Pathological glucose metabolism affects the function and viability of osteoblasts, osteoclasts and osteocytes, which are all the major effector cells in bone remodeling. Osteoblasts and osteoclasts express a receptor for AGEs, which was found to be up-regulated in conditions of hyperglycemia [237]. Hyperglycemia and AGEs together suppress proliferation, differentiation and function of osteoblasts, possibly via down-expression of BMPs and Runx2 as well as activation of PPAR-γ signaling [154]. In vitro studies further found that a couple of pathways are involved in a hyperglycemia-induced negative impact on osteoblasts, including STAT3/SOCS3, PI3K/Akt, EphB4/EphrinB2, NO/cGMP/PKG signaling pathway [155]. Hyperglycemia and AGEs could also suppress bone anabolism indirectly by stimulating the production of osteocyte-derived sclerostin. Furthermore, AGEs hamper the survival of osteoblasts and osteocytes [156]. The impact of hyperglycemia on biomineralization remains controversial. Variations in glucose concentration, incubation condition and cell lineages among in vitro studies might account for the discrepancy. Emerging evidence supports that high glucose concentration causes an increased rate of bone formation but the poor quality of mineralized tissue [155]. Nevertheless, no agreement has been reached about how exactly impaired glucose homeostasis affects osteoclasts. In osteocyte-like MLO-Y4-A2 cells, high levels of glucose and AGEs lowered the production of RANKL, which might account for inhibited osteoclastogenesis [156]. They also reduce the autophagy level of osteoclasts and result in cellular dysfunction [157]. Intriguingly, in RAW264.7 cell-derived osteoclast-like cells, AGEs modulate bone resorption in an exposure-time-dependent pattern. Bone resorption activity is suppressed initially but enhanced in the later stage during exposure to AGEs [158]. Similarly, a diabetic rat model induced by streptozotocin suggests that diabetes mellitus elevates osteoclast numbers and bone resorption activity [159]. Conflicting findings about osteoclastic bone resorption from in vitro and in vivo studies might be a hint that not only high glucose concentration, but also other pathophysiological conditions that are secondary to disordered glucose metabolism deserve attention. These conditions include hypoxia-induced local acidosis, an excess of ROS and activation of Ca^2+^/calmodulin-dependent protein kinase II in diabetes mellitus [159,160].

Under conditions of hyperglycemia, impaired periodontal anabolism resembles that of systemic bone, during which process PDL stem cells matter a lot. Hyperglycemia suppresses the transcription of Runx2 and SOX9 in periodontal tissues, representing a decreased level of osteoblastic differentiation [161]. Under conditions of systemic high glucose, the over-activated AGEs-AGE receptor pathway down-regulates expression of osteogenic genes expression (ALP, BSP, osteopontin, and Runx2) in human PDL stem cells via PKC phosphorylation [162]. Enhanced DNA methylation also contributes to hyperglycemia-inhibited osteogenic differentiation in human PDL stem cells [163]. Furthermore, cell proliferation of human PDL stem cells is also impaired by hyperglycemia [155]. Hyperglycemia favors osteoclastic resorptive activity in periodontal tissues. The level of glycated hemoglobin, an important indicator of glucose homeostasis, is in direct correlation with OPG. RANKL/OPG ratio in hyperglycemic individuals is higher than in nondiabetic controls [164]. In hyperglycemic state, osteoclasts exhibit augmented fusion and higher osteoclastic activity but osteoclasts show lower sensitivity to LPS stimulation, preventing osteoclasts from deactivation [165]. It is an intriguing finding that occlusal trauma, a secondary phenomenon of periodontitis in some cases, along with hyperglycemia might exacerbate periodontal bone destruction via enhanced expression of CSF-1 and VEGF in human PDL [155]. Notably, osteocyte also occupies a central position in hyperglycemia-associated periodontal bone loss. High glucose stimulates the increment of MiR-124-3p carried by osteocyte-secreted exosomes, which further suppresses osteoblast formation via down-regulating galectin-3 [166]. Dysregulated glucose metabolism elevates AGE levels in GCF [167]. AGEs and *P. gingivalis* LPS synergistically curb osteoblastic bone formation via upregulating osteocyte-derived sclerostin [168]. Moreover, ROS and TNF-α are suggested to promote sclerostin expression in osteocytes [169]. Osteocyte also facilitates alveolar bone loss in diabetic periodontitis by acting as a principal source of RANKL for osteoclastogenesis [170].

Current literature also indicates that hyperglycemia further modulates local flora and host immune-inflammatory response, indirectly modifying cellular behaviors of periodontal cells. Oral microbiome studies demonstrate that glycemic dysregulation of diabetic patients alters the composition of the periodontal microbiota, in a glucose-concentration and periodontitis-stage associated pattern [171]. At the same time, the expression of pathogen receptors is also elevated under hyperglycemic conditions [172]. Periodontal immune-inflammatory components, including cells and cytokines, are affected by glycemic dysregulation. Chronic hyperglycemia increases the AGE level in GCF and periodontal extracellular matrix, facilitating inflammatory infiltration via the AGE/AGE receptor pathway, and in turn, inflammation exacerbates matrix glycation and AGEs deposition in periodontal tissues [173]. Furthermore, abnormal glucose metabolism modulates the functional activity of critical inflammatory cells, such as neutrophils, monocytes and macrophages [154]. Macrophages have newly been identified as essential effector cells of hyperglycemia-related inflammation and tissue destruction in the periodontal micro-environment and a plethora of potential pathways are gradually recognized. First, in an inflammatory state, hyperglycemia excessively enhances ROS production, consequently tipping the balance of macrophage polarization towards the M1 phenotype, a pro-inflammatory subtype [174]. Second, macrophage-mediated inflammation and senescence are enhanced under hyperglycemic conditions, contributing to sustained inflammation status and aggravated periodontal damage [175]. Third, hyperglycemia-triggered macrophage pyroptosis associates with periodontal fibroblast senescence possibly via phosphorylation of NLR family CARD domain-containing protein 4 [176]. At last, immunocompromised macrophages in hyperglycemia bring about uncontrollable infection [177]. In addition to those inflammatory cells, the altered inflammatory profile is also observed in both rat models and humans with diabetic periodontitis. This profile is characterized by increased pro-inflammatory cytokines [161] (e.g., TNF-α, IL-1β, IL-6, IL-4, IL-10, IL-17), reduced anti-inflammatory proteins [164] (e.g., IL-10, FGF-21, monocyte chemotactic protein-1, TNF-β). Moreover, NLRP3 inflammasome [242], inducible nitric oxide synthase [243], CC chemokine ligand 2 [244], C3 [245], IL-17 [246] are proposed as essential mediators in diabetes mellitus-enhanced periodontal damage.

Historically, the relationship between glucose metabolism and bone remodeling has been regarded to be unidirectional. Recent studies shed light on the influence of osteoporosis on glucose homeostasis. In OVX rats, enhanced bone resorption and bone mineral density decline caused high blood glucose levels [247]. Several signaling molecules and metabolites of bone remodeling have been found to regulate glycemic homeostasis. Undercarboxylated OCN released into serum during the process of bone matrix degeneration may improve glucose tolerance [248]. Osteoclast-derived dipeptidyl peptidase 4, as an osteoclast-osteoblast coupling factor during bone remodeling, possibly helps improve glucose homeostasis [249]. Osteoblasts also participate in glucose metabolic regulation via FoxO1, a critical transcriptional factor in the regulation of glycemic metabolism [250].

#### 3.2.2. Lipid Metabolism

A broad category of biomolecules that consist of FAs is collectively referred to as lipids. FAs could be divided into the following two groups: saturated FAs (SFAs) and unsaturated FAs (UFAs). Lipids exert essential effects in many physiological processes, including energy metabolism, cellular communication and cell membrane assembly [251]. Hyperlipidemia, an aberrant condition of systemic lipid metabolism, commonly manifests as elevated serum levels of triglyceride, total cholesterol, low-density lipoprotein (LDL) and decreased circulating high-density lipoprotein (HDL) [252]. Adipose tissues, in which adipocytes are the most abundant host cells, are the major lipid reservoirs to store excess triglyceride in blood [251]. In bone marrow milieu, the accumulation of excessive lipids manifests as bone marrow adiposity.

##### Dyslipidemia with Osteoporosis and Periodontitis

Disorders of lipid metabolism either in serum (hyperlipidemia) or bone micro-environment (bone marrow adiposity) have been suggested as risk factors for bone metabolic disorders, such as osteoporosis [178]. Epidemiologic investigations indicate that low HDL levels, high LDL and total cholesterol levels in serum play critical roles in the occurrence of senile, postmenopausal and diabetes mellitus-related osteoporosis [179,180]. Sivas F et al. revealed that lipid level in circulation significantly correlates with the prevalence of osteoporotic fracture, but not bone mineral density change [181]. Remarkably, a class of lipid-lowering drugs, statins, have been proven to protect bone from osteoporotic bone loss [182]. Considering their capability of acting on bone directly, caution is still needed when determining whether the protective effects of statins in bone are attributed to lipid-normalizing actions. A cyclic relationship between bone marrow adiposity and osteoporosis has also been established. Epidemiological and animal studies demonstrate that osteoporosis is evidently correlated with increased fat content and altered lipid composition of bone marrow [183]. Additionally, in human bone marrow supernatant fluid, tandem mass tag-based proteomics analysis suggests a causal link between lipid metabolism and osteoporosis. This association is evidenced by differentially expressed perilipin-1, a regulator of lipid metabolism, in osteoporotic populations [184]. In turn, chronic osteoporosis also induces a specific change in lipid profile in bone marrow and mineralized tissue. Abnormal levels of TG, cholesteryl esters, sphingomyelin, stearoyl-CoA desaturase and free FAs, which are indicative of perturbated lipid metabolism, were detected in OVX rats [178].

Reciprocal actions present between impaired lipid metabolism and periodontitis as well. Hyperlipidemic individuals manifest advanced periodontal inflammation and tissue destruction compared to normolipidemic subjects [185]. In the meantime, periodontitis alters the serum lipid profile in a disease severity-dependent manner [253] and periodontal therapy helps improve the profile of blood lipids [254]. Nevertheless, these epidemiological studies could not support the cause–effect relationship between hyperlipidemia and periodontitis due to the limitations of cross-sectional studies. The results of animal studies seem to be conflicting. Rats on a cholesterol-enriched diet showed increased total cholesterol, LDL and HDL in circulation. This hyperlipidemia rat model demonstrates that a high-cholesterol diet induces spontaneous alveolar bone destruction but exhibits no addictive effect on periodontitis-induced bone loss [186]. On the opposite, some other studies found that obesity/hyperlipidemia induced by high fat and hypercaloric diet act as potentiating factors for ligature-induced periodontal destruction in rats [187]. Variations of diet composition, induction methods of periodontitis and timespan of experiment probably contribute to the inconsistency between results of animal studies.

Mechanisms underpinning the interactions between abnormal lipid profiles and bone metabolism are complex. Lipids participate in bone metabolism by acting as signaling molecules. Cellular behaviors of osteoblast lineages are impaired by hyperlipidemia [188]. Diet-induced hyper-cholesterolaemia exerts detrimental effects on the proliferation and differentiation of mouse osteoblasts in a concentration-associated pattern [189]. Furthermore, high plasma cholesterols accelerate osteoclastic bone resorption. Efficient cholesterol delivery into osteoclasts by LDL is essential for cellular survival. Conversely, cholesterol insufficiency impairs cell function by inhibiting the activity of vacuolar-type ATPase and induces silencing of survival signaling in osteoclast [190].

Bone marrow adipose tissues exert hazardous effects on bone in a paracrine and endocrine pattern via releasing a large body of regulatory factors, including adipokines, free FAs, and pro-inflammatory mediators [191]. Studies focusing on how adipokines modulate phenotypes of critical bone cells have been performed extensively [150]. In a dyslipidemic rat model, increased chemerin, an adipokine, contributed to enhanced osteoclast resorption activity and bone loss [192]. Adiponectin, an adipose tissue-derived factor, modulates polarization and infiltration of macrophages and attenuates alveolar bone loss in periodontitis. Nevertheless, obese status leads to the down-expression of adiponectin [193]. Adipokine leptin participates in the regulation of bone metabolism directly via activating leptin receptors on osteoblasts and indirectly via activating sympathetic signaling [194]. Bone metabolism-related neural pathways will be elucidated in the chart of “psychological stress”.

In the context of periodontitis, mechanistic investigations found that dysregulated lipid metabolism enhances pathogen loads, inflammation and oxidative damage. The prevalence of periodontal pathogens, including *Fusobacterium nucleatum* and *P. intermedia*, was elevated in rats fed a fat-enriched diet compared to rats fed a normal chow [195]. In terms of inflammatory burdens of the host, Cavagni J et al. reported that hyperlipidemia does not bring about significant changes in IL-1β and TNF-α [196]. On the contrary, Montalvany-Antonucci CC et al. proposed that expressions of 30 inflammatory genes in alveolar bone are regulated by a high-fat diet [197]. Kırzıoğlu FY et al. found that food rich in cholesterol leads to hyperlipidemia, and then promotes the infiltration of polymorphonuclear leukocytes [186]. A hyperlipidemic rabbit model with experimental periodontitis was established by Chen S et al. to explore the exact impact of hyperlipidemia on host response to bacteria and it was found that the actions vary with different phases of infection. The inflammatory response in rabbits on a fat-enriched diet is weaker in the early stage and stronger in the long term than in those on a normal diet. The augmented inflammatory response in chronic periodontitis probably relates to accumulated inflammatory cytokines during sustained infection, and further contributes to alveolar bone loss [198]. Lipid oxidation is also reported to participate in the development of periodontitis [186]. In rats with ligature-induced periodontitis, augmented lipid peroxidation and inhibited functions of the endogenic antioxidative system were detected [199]. In line with animal studies, clinical investigations also demonstrated that increased oxidation products of lipids and oxidative DNA damage are likely to be involved in the cross-talk between periodontitis and hyperlipidemia [200,201]. In vitro experiments indicated that oxidized LDL activates the NF-κB pathway and further stimulates the release of pro-inflammatory cytokines, including IL-8, IL-1β and PGE2 in human gingival epithelial cells [202]. It was also reported that oxidized lipids inhibit osteoblast differentiation, whereas they promote the immigration and differentiation of osteoclast progenitors in vitro [203]. It is intriguing that besides hyperglycemia, a high level of lipids (triglyceride and LDL) is also an important prerequisite for the synthesis of AGEs [204]. The comprehensive effects of AGEs on periodontitis have been elucidated in the Section 3.2.1. It is worth noting that periodontal infection could aggravate lipid metabolic disorder, and in turn potentiate periodontal bone destruction. The invasion of periodontal pathogens, mostly Gram-negative bacteria, induces the release of various inflammatory cytokines, including IL-1 and TNF-α. Joint actions of these cytokines further increase free FAs, LDL and TG in circulation. Moreover, LPS could combine with LDL and further prevent the hydrolysis of LDL [205]. Activation of LPS-TLR2 signaling upregulates the expression of the receptor for oxidized LDL in rat bone marrow macrophages. The amplified signaling of oxidized LDL thereafter assists LPS in promoting osteoclastogensis [206]. Periodontal pathogens are suggested to stimulate uptake and inhibit efflux of lipids in macrophages, rendering lipids accumulation in cells [207]. Since osteoclasts are derived from monocyte/macrophage lineage, it is plausible to speculate that similar phenomena are present in osteoclasts as well. Considering that insufficiency of intracellular lipids impairs the function and viability of osteoclasts, it is likely that periodontal microorganisms contribute to alveolar bone resorption via modulating lipid homeostasis in osteoclasts.

##### FAs Profile with Osteoporosis and Periodontitis

FAs emerge as another potential link between lipid metabolism and bone metabolism. It has been found that the composition of free FAs in bone marrow sera varies in different states of bone metabolism. In the stage of osteoporosis development, osteoclast-mediated bone resorption dominates bone remodeling. After the occurrence of osteoporotic fracture, osteoblastic bone formation is enhanced in support of the need for repair. In osteoporotic individuals without fracture, the ratio of saturated to unsaturated free FAs in bone marrow sera is higher than that in circulation. However, the ratio decreased after osteoporotic fracture [183].

Under the context of abnormal lipid metabolism, an altered FAs profile emerges as a potential contributor to the clinical occurrence of periodontitis [208]. Palmitate aggravates *P. gingivalis*-triggered periodontal inflammatory response and alveolar bone destruction in C57BL/6 mice [209]. An animal model with *P. gingivalis*-induced periodontitis indicated that distinct from exacerbation of palmitic acid on periodontal destruction, oleic acid brought little change to alveolar bone metabolism under the inflammatory condition when compared with controls on a normal caloric diet [210]. Moreover, in hypercholesterolemic rats with ligature-induced periodontitis, replacing an SFAs-enriched diet with a diet rich in omega-3 poly-UFAs resulted in mitigated alveolar bone loss [211].

In support of clinical findings, accumulating mechanistic data indicate that the saturation degree determines disparate effects of FAs on the viability and functional activities of bone cells. Generally speaking, SFAs suppress bone formation but facilitate bone resorption. UFAs curb osteoclastic bone resorption. Such cell-specific regulations of autophagy and apoptosis contribute to bone impairment in the presence of a high concentration of palmitic acid (a kind of SFA) as opposed to a hyperlipidemic level of oleic acid (a kind of UFA) [178,191]. Thereinto, the hyperlipidemic level of palmitic acid was reported to exert an inhibitory effect on osteoblastic mineralization activity and bone formation [212]. C16-ceramide accumulation, which presented in the serum of rats on the palmitic acid diet, may link palmitic acid with osteoblast dysfunction via apoptosis [213]. Palmitic acid further enhances the development and function of osteoclasts [214]. Contrary to SFAs, UFAs inhibit the formation and activity of osteoclasts regardless of chain lengthening [190]. PPARs are a family of nuclear receptors dominating the regulation of lipid metabolism and bone homeostasis. Upon binding to and activating PPARs, Poly-UFAs then restrain osteoclastogenesis [190]. Moreover, in both cultures of human and murine osteoblast cell lines, poly-UFAs supplementation, omega-3 poly-UFAs in particular, exerts a protective effect on osteoporosis via modulating the production of PGE2 [215]. However, the mechanism underlying the impact of perturbed FAs profile on bone metabolism from the perspective of energy metabolism awaits further exploration.

In addition to bone cells, periodontal effects of FAs are further based on pleiotropic actions on resident cells and immune components, including cytokines, lymphocytes, the natural killer cells and phagocytosis [205]. Remarkably, similar to saturation-dependent actions on bone cells, the effects of FAs on fibroblasts and the inflammatory response of periodontal tissues are distinct between SFAs and UFAs. On one hand, SFAs and UFAs were also reported to exert disparate effects on PDL fibroblasts. Palmitic acid curbs cellular survival, while oleic acid enhances osteogenic differentiation in PDL fibroblasts [216]. On the other hand, SFAs augment periodontal inflammation, while UFAs are reported to be anti-inflammatory agents. The interaction of SFAs, such as palmitic acid, stearic acid and arachidonic acid with FAs translocase (CD36) enhances periodontal inflammation via the activated TLR signaling pathway [210]. The expression of CD36 in periodontal tissues was detected to be elevated by the synergetic effects of periodontitis and a high-fat diet [217]. Palmitate-CD36 combination further increased the production of cytokines and chemokines, as well as elevated infiltration of critical inflammatory cells into the periodontitis niche, including monocytes and neutrophils [209]. Different from SFAs, the biological actions of UFAs are mediated by another FA membrane receptor, GPR120 [218]. Furthermore, in the culture of human gingival fibroblasts, omega-3 poly-UFAs, such as docosahexaenoic acid and eicosapentaenoic acid, and their derivatives seem to suppress the palmitic acid-induced release of inflammatory cytokines, IL-6 and IL-8 [219]. Additionally, immune cells are modified by UFAs. UFAs suppress lymphocyte proliferation via mitogenesis-attenuation and modulation of cytokines production and enhance cellular phagocytosis to remove pathogens. Some UFAs even restrain activity of the natural killer cells [205].

#### 3.2.3. Amino Acid Metabolism

AAs refer to a group of organic molecules, which are characterized by the concomitant presence of both amino and acid groups. Twenty AAs that could act as building blocks of protein or polypeptide are typically categorized as essential and non-essential AAs. AAs possess pleiotropic properties in various cellular processes, especially energy metabolism. Disordered AAs metabolism has been extensively studied and well-understood in the fields of neurological, cardiovascular and oxidative stress-related disorders [255]. However, whether and how AAs metabolism impacts bone homeostasis have never been comprehensively elucidated. In this part, we aim to provide a novel insight into the role of AAs profile in bone metabolism disorders, osteoporosis and periodontitis.

Epidemiological, animal and metabolomics studies support the connection between osteoporosis and AAs profile, including the pivotal roles of some AAs in bone homeostasis [220,221]. BCAAs, aromatic AAs, alanine, glycine, and proline protect bone health, while deficiency of them increases susceptibility to osteoporosis [221,222,223,224,225,226]. Individuals with high serum levels of total homocysteine, a sulphur-containing AA, were observed to be more vulnerable to bone mineral density reduction and osteoporotic fracture [221].

However, the relationship between periodontitis and AAs profile is scarcely studied. Clinical studies reveal that the composition of salivary-free AAs varies between periodontitis patients and healthy counterparts. Moreover, several AAs, including methionine, citrulline, carnosine, and arginine, are in an obvious and positive correlation with inflammatory markers of periodontal disease [256]. It is noteworthy that the AAs profile between GCF and saliva was found to be quite different. Therefore, which type of oral fluids should be chosen ought to be determined before exploring the relationship between free AAs signature and periodontitis.

Distinct roles of different AAs in bone homeostasis are further revealed by mechanistic explorations. Sulphur-containing (methionine, cysteine) AAs accelerate bone loss by suppressing osteoblast functions and enhancing osteoclast activity, which might be accredited to increased acid load during the oxidative metabolism of sulphur-containing AAs [221]. Aromatic AAs (tryptophan and tyrosine) supplementation promotes the proliferation and osteogenic differentiation of bone marrow MSCs, which probably associates with elevated circulating IGF-1 [227]. In a culture of C57BL/6 mice bone marrow MSCs, oxidized aromatic AAs, including di-tyrosine and kynurenine, almost block the anabolic actions of aromatic AAs on bone and the antagonistic action of kynurenine is particularly significant [228]. Many other derivatives of tryptophan, including melatonin and serotonin, also actively participate in the regulation of bone remodeling. Melatonin promotes bone anabolism as we elucidated previously. The regulation of serotonin in bone remodeling depends on its source. Serotonin derived from the brain benefits bone health, while serotonin synthesized in the gut suppresses bone formation [257]. Deficient transport of selective cationic AAs, including lysine, arginine and ornithine, is the hallmark of lysinuric protein intolerance. Patients with lysinuric protein intolerance were reported to be at high risk of osteoporosis. Impaired synthesis of bone matrix protein and accelerated turnover of collagen are suggested to be the part of underlying mechanisms of osteoporotic change in those individuals [229]. Meanwhile, there might be other potential pathways besides disturbed collagen metabolism. In vitro, arginine and lysine boost the proliferation, differentiation and functional activity of osteoblasts that are derived from osteopenic bone [230]. Arginine supplementation is capable of stimulating the activation of the GH/IGF-1 axis and the synthesis of nitric oxide. The GH/IGF-1 axis enhances bone anabolism, and nitric oxide inhibits osteoclastic bone resorption [231]. Oral administration of L-lysine increases absorption and conservation of calcium, respectively, in the gut and kidney. It is plausible that calcium homeostasis links lysine with bone homeostasis [232]. Notably, the creatine/phosphorylcreatine system participates in cellular energy metabolism and a previous review demonstrated that creatine supplementation affects bone remodeling in the elders. However, the exact impact of creatine on bone metabolism and the potential mechanism still awaits further exploration [258]. Specific AAs profiles further link various pathological conditions with osteoporosis. Autophagy, a cellular process of recycling proteins and organelles, is critical for the maintenance of AA homeostasis in cells. Dysfunctional autophagic machinery is a potential etiologic factor for abnormal AAs profile. A large body of evidence reveals that aberrant autophagy and AA metabolism are associated with osteoporosis [226]. Furthermore, disturbed AA metabolism was found to be a potential mediator of gut dysbiosis-induced osteoporosis [233].

Evidence with regard to the role of AA metabolism in periodontal pathology is limited and primarily centers on the actions of oral bacteria. AAs, rather than glucose or sucrose, are the main energy substrates for red complex species in the periodontal pocket, namely, *P. gingivalis*, *Tannerella forsythensis* and *Treponema denticola* [234]. Lysine and arginine provide energy for *Eubacterium nodatum*, an anaerobe in the human periodontal pocket [235]. In addition, specific AAs derived from the biosynthesis of gut microorganisms tend to influence AAs homeostasis in the host, and similar observations have also been reported in oral microflora as well [236,256].

## 4. Lifestyle

**Figure 4:**
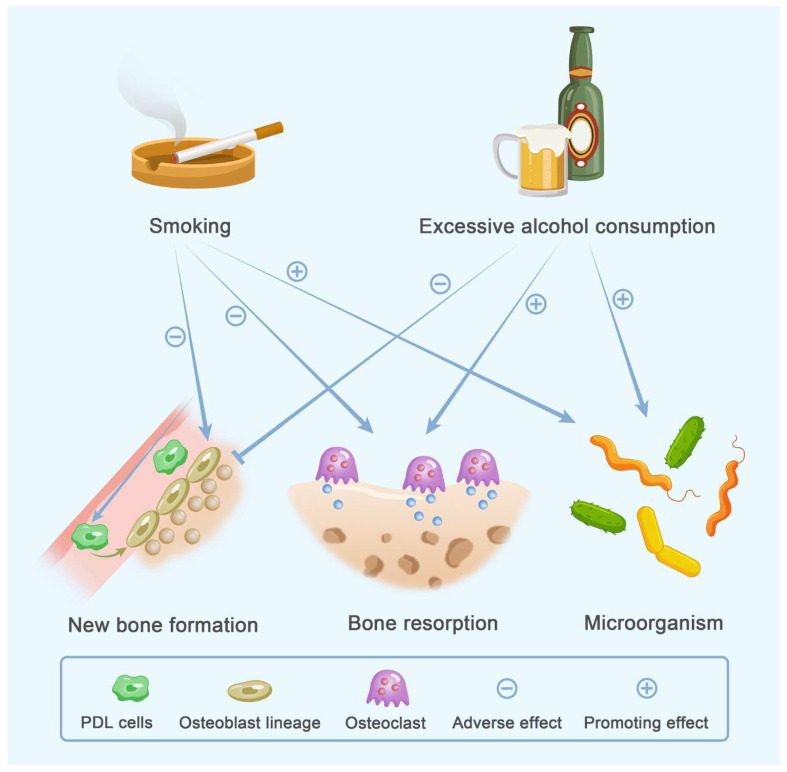
Impacts of lifestyle on periodontal status. Smoking and excessive alcohol consumption are typical unhealthy lifestyles. They inhibit the osteogenic differentiation of PDL cells and osteoblastic bone formation and promote osteoclastic bone resorption. Furthermore, they also aggravate the bacterial infection. PDL, periodontal ligament.

It is well-accepted that there is a close relationship between osteoporosis and lifestyle, including nutritional factors and behavioral factors. The majority of nutritional factors have been elaborated on above, such as calcium, vitamin D and energy substrates. Herein, we focus on how behavioral factors, including excessive drinking and smoking, influence bone metabolism (Figure 4) (Table 3). Immoderate consumption of alcohol and cigarette has been proven to impair bone health and elevate vulnerability to osteoporotic fracture [259]. Specifically, in contrast to smoking as an evident predisposing factor for osteoporosis, the role of alcohol consumption in osteoporosis development is equivocal [260].

### 4.1. Smoking

Cigarette smoking has been verified to increase osteoporotic fracture risk in both sexes [261]. Smoking has been recognized as an important predisposing factor for the initiation and progression of periodontitis [262]. Periodontal destruction, including alveolar bone loss, is in proportion to the duration and frequency of tobacco use [263]. There is a special form of smoking, secondhand smoking, which has been also found to deteriorate periodontal health [264]. Data from the Fourth and Fifth Korea National Health and Nutrition Examination Surveys implicate that despite weaker prediction ability than active smoke, secondhand smoke is still a potential predictor for periodontitis risk [299]. More importantly, smoking-induced intensification of periodontitis, including the occurrence, severity and progression and outcome of the disease, could be reversed by smoking cessation to some extent [300].

The harmful effects of smoking on bone health have been extensively investigated in mechanistic studies and could be classified into direct and indirect actions. It is suggested by a genome-wide meta-analysis that smoking-induced DNA methylation is a prospective mechanism of tobacco-related diseases, including osteoporosis [265]. Among over 7000 chemicals in cigarette smoke, some gradients, like nicotine, cadmium, polycyclic aryl hydrocarbons and dioxins, exert direct deleterious effects on bone. Nicotine has received the most attention in past decades. Nicotine acts with a nicotinic receptor on osteoblasts to regulate cellular proliferation at the transcriptional level in a biphasic manner. The proliferation and even viability of osteoblasts are inhibited by a high level of nicotine [266]. The in vitro experiment indicates that nicotine directly elicits the differentiation of human osteoclast precursors preliminarily, while the formation of mature osteoclasts with resorption function afterwards requires the concomitant presence of M-CSF and RANKL [267]. Aside from nicotine, cigarette smoke also contains polycyclic aryl hydrocarbons and dioxins. They are both ligands for AhR expressed on osteoblasts and osteoclasts. Activation of AhR further promotes the formation and activity of osteoclasts via the AhR-c-Fos pathway [268]. In the meantime, upon binding with its ligand, AhR displays an inhibitory effect on osteoblastic differentiation in a dose-dependent pattern [269]. Moreover, the effect of cadmium on bone has gradually been recognized. In a Swedish cohort of male elders, exposure to cadmium during smoking was found to make up approximately half of the gross effect of tobacco-induced bone mineral density decline [270]. In addition to those direct actions on bone, smoking also inhibits calcium resorption and impairs vitamin D metabolism. Smoking further elevates serum levels of cortisol and free radicals, and reduces circulating estradiol [261].

As an evident behavioral risk factor for periodontitis, tobacco smoking impacts periodontal tissues in various aspects [271], including periodontal bone cells, local microbiome, host immune defense, and redox homeostasis. Aside from phenotypic alternation revealed by clinical, animal and cellular evidence, tobacco smoke modulates immunological response, bone metabolism and tissue healing at the genetic level, causing a genetic predisposition to periodontal disease [301].

Periodontitis-related tissue destruction, alveolar bone loss, in particular, is aggravated by exposure to tobacco smoke [272]. It has been reported that the receptor for nicotine is up-expressed in both PDL tissues and PDL stem cells under inflammatory conditions [273]. Nicotine absorbed by oral mucosa displays stimulatory action on periodontitis-induced osteoclastic bone resorption, which is mediated by increased IL-1β in the periodontal niche [274]. Nicotine also promotes MMP-mediated collagen degeneration, which subsequently modulates osteoclast behaviors (migration and adhesion) and induces osteoblast death [275]. Moreover, nicotine directly stimulates the differentiation of osteoclast progenitors as we illustrated previously [267].

Smoking suppresses the regeneration of periodontal tissues, especially alveolar bone. Nicotine decreases the viability of PDL fibroblasts via the up-regulated activity of cellular autophagy [276,277]. Nicotine also exhibits cytotoxicity on periodontal MSCs in a dose and duration-dependent manner [278]. Smoker PDL-derived stem cells display suppressed proliferation, migration and osteogenic differentiation compared to controls from a non-smoker. The elevated expression of nicotine-associated microRNAs possibly mediates smoke-induced impairment to regenerative potentials of PDL stem cells [279]. Additionally, benzo[a]pyrene (polycyclic aryl hydrocarbons) inhibits the capability of PDL cells to differentiate into osteoblasts and synthesize collagen, and the inhibitory effect could be reversed by the blockage of AhR [280]. Apart from local MSCs, circulating MSCs are also modulated by tobacco smoke and nicotine, manifested by reduced number, suppressed homing and function of MSCs [281]. At last, as a vasoactive molecule, nicotine contributes to decreased periodontal vascularization under the condition of periodontitis, which probably results from the smoke-induced decline of angiogenesis-related proteins [282].

Cigarette smoke induces subgingival microbial dysbiosis independent of periodontal status [283]. In response to the stimulus of tobacco smoke, the formation rate and virulence of subgingival biofilm are promoted by several critical pathogens, especially *P. gingivalis* and *F. nucleatum.* At the same time, both innate and adaptive host immune defenses are compromised by smoking [284]. For instance, the immigration and phagocytic function of neutrophils in periodontal tissues are restrained when expose to tobacco smoke [275]. Tobacco consumption also alters the profile of salivary antimicrobial peptides [285]. Chronic nicotine administration enhances periodontal destruction in a rat model with ligature-induced periodontitis possibly via activation of the cholinergic anti-inflammatory signaling pathway [286]. Eventually, the balance between host defense and bacterial invasion is disturbed, or the pre-existing imbalance is exacerbated. Furthermore, tobacco consumption also disrupts oxidative balance, further aggravating periodontitis [287].

### 4.2. Alcohol Consumption

Despite the influence of alcohol on osteoporosis susceptibility being far less significant than that of smoking as we described before, clinical evidence still supports that alcohol drinking indeed damages bone health. Recent literature demonstrates that alcohol consumption positively correlates with osteoporosis susceptibility, and the risk is in proportion to the daily intake of alcohol [288]. Some studies suggest that the correlation between alcohol intake and fracture risk is non-linear, which reveals a “J” shape correlation curve. In other words, light/moderate intake of alcohol tends to reduce hip fracture risk, while heavy alcohol intake is in a close relationship with bone deterioration and high fracture risk [289]. However, when assessing density, micro-architecture and geometry of the distal radius and tibia by high-resolution computed tomography, measurement data suggest that light drinking deteriorates bone health as well. Differences in demographic characteristics of samples (such as age and systemic status), duration and extent of alcohol consumption among studies might contribute to the inconsistency [302]. A mice model with experimental chronic alcohol consumption exhibits a marked decline in bone mineral density after 4 weeks of alcohol administration.

There is a linear relationship between the dose of alcohol consumption and the occurrence/severity of periodontitis, especially alveolar bone loss. However, ingestion of a small amount of alcohol does not exert much adverse effect on periodontal tissues [290]. Mendelian randomization, an approach to make a casual inference, has been applied to determine the role of alcohol consumption in the development of periodontitis and the result supports that alcohol ingestion is a predisposing factor for periodontitis [291]. In addition, the correlation between alcohol intake and periodontitis was reported to be gender-specific by some studies, which suggests more significant relevance in male than in female populations [292]. Corroborating with clinical findings, light alcohol intake does not aggravate ligature-induced damage of alveolar bone and even seems to protect against bone loss at unligated sites in rats [293]. Heavy and chronic alcohol consumption causes spontaneous alveolar bone destruction in rats without pre-existing periodontitis and a high frequency of alcohol intake causes more severe bone loss than occasional consumption [294]. Nevertheless, another study suggests that ethanol displays no impact on the periodontal bone of unligated sites regardless of ethanol concentration in a rat model with ligatured-induced periodontitis [295]. Intriguingly, the caloric value of alcohol has been found to be a non-negligible contributor to drinking-associated periodontal bone loss. Thus, only when compared with animals on a diet of isocaloric amounts of glucose rather than water (traditionally), the exact effect of ethanol itself on periodontal bone destruction could be learned. In this case, alveolar bone loss of an unligated tooth is unchanged, even decreased slightly after the elimination of the caloric factor [296]. At ligated sites, alcohol binge consumption, in other words, intensified and intermittent alcohol intake, exacerbated the periodontitis-induced deterioration of alveolar bone, evidenced by reduced bone mineral density, thickness and number of bone trabecula [296]. Alcohol intake was found to aggravate periodontal inflammatory response and osteoclastic bone resorption [297]. Although exacerbation of heavy alcohol intake on periodontitis-related bone loss of ligated teeth is well accepted, agreement on the dose–effect relationship has not been achieved yet [295,297]. Furthermore, in rats with LPS-induced periodontitis, excessive alcohol intake does not contribute to the amplification of installed disease and alveolar bone loss [298]. To clarify this conflicting result, a comprehensive understanding concerning how alcohol influence bacteria and inflammatory response is needed.

Importantly, the deleterious effect of excessive and long-term alcohol consumption on bone is evident. Mechanistic evidence suggests that direct actions on bone cells and other indirect actions cumulatively contribute to the development of alcohol-related osteoporosis. Alcohol consumption modifies the cellular behaviors of bone effector cells. Alcohol administration inhibits bone anabolism, evidenced by the declined number and function of osteoblasts [302]. In alcohol administrated-human bone marrow MSCs, activation of the TNF-α signaling pathway due to enhanced endoplasmic reticulum stress impairs osteoblastogenesis of MSCs [303]. In a mouse model with chronic and excessive alcohol intake, lineage commitment of bone marrow MSCs is altered towards adipogenic differentiation rather than osteoblastic differentiation. Activation of the PI3K/AKT/mTOR signaling pathway caused the down-expression of Runx2 and up-expression of PPARγ, underlying the mechanism of disturbed adipo/osteogenic differentiation of rat bone marrow MSCs [304]. Except for PI3K/AKT/mTOR signaling, many other signaling pathways, including Wnt/β-catenin, FoxO, TGF-β and BMP signaling, have been recognized to participate in impaired osteogenic differentiation of MSCs in response to heavy alcohol drinking [305]. Premature cellular senescence of human-derived bone marrow MSCs are proportionally correlated with ethanol dose, and this possibly also contributes to decreased osteogenic potential under the context of alcohol exposure [306]. An excess of acetaldehyde, the main product of alcohol oxidative metabolism, suppresses the proliferation, differentiation and survival of osteoblasts [307]. Similarly, DNA synthesis and cell proliferation are inhibited by alcohol administration in cultures of human osteoblast-like osteosarcoma cells [308]. Moreover, the production of bone structural proteins is declined in rats fed on a long-term alcohol diet [309]. In humans and rodents, the differentiation and activity of osteoclasts are enhanced under the condition of alcohol consumption [305]. Alcohol elevates the production of ROS, which induces the activation of ERK/STAT3 signaling and up-regulated expression of RANKL in osteoblasts [305]. A mice model with experimental chronic alcohol consumption indicates that long-term alcohol intake inhibits the function of antigen-presenting cells and the natural killer T-like cells, further decreasing the production of IL-4, a potent inhibitor of osteoclastogenesis. On the contrary, two mediators of osteoclastic differentiation, NFATc1 and RANKL, are up-expressed [310]. Other osteoclastogenic cytokines, including IL-6, IL-1β and TNF-α, are also increased under the condition of heavy alcohol drinking [311]. The inhibitory effect of these three cytokines on osteoblastogenesis are gradually recognized recently [312]. In addition to osteoblasts, osteoclasts and their precursors, osteocytes are also impaired by alcohol. Alcohol exposure enhances the apoptosis of osteocytes, resulting in bone mineral density decline [313]. Similar to smoking, alcohol intake exhibits various indirect actions as well, bringing about endocrine changes that are detrimental to bone homeostasis. Under the condition of heavy alcohol intake, the production of sex steroids, GH/IGF-1 and cortisol are decreased [314]. Perturbed calcium homeostasis by immoderate alcohol consumption via modulation of vitamin D and PTH has been reported as well [313]. Additionally, chronic and excessive alcohol intake increases the production of ROS [312] and shifts the composition of intestinal flora towards a pro-inflammatory profile [315]. In contrast to heavy consumption, mechanistic knowledge with regard to the influence of light/moderated alcohol drinking on bone is scarce. Some studies suggest that consuming a small amount of alcohol increases the level of calcitonin and estrogen, which helps explain the bone protective effect of light/moderate alcohol consumption [313].

Alcohol dependence alters the composition of oral micro-flora and elevates the levels of specific subgingival pathogens, including *P. intermedia*, *E. corrodens* and *F. nucleatum* [316,317]. Alcohol abuse in men compromises the neutrophil function of bacterial killing at sites without periodontitis, while the neutrophil function at sites with pre-existing inflammation is enhanced, contributing to tissue destruction [318]. Disturbed redox homeostasis by alcohol and its toxic metabolites also accounts for alcohol-aggravated periodontitis [319].

## 5. Psychological Stress

**Figure 5:**
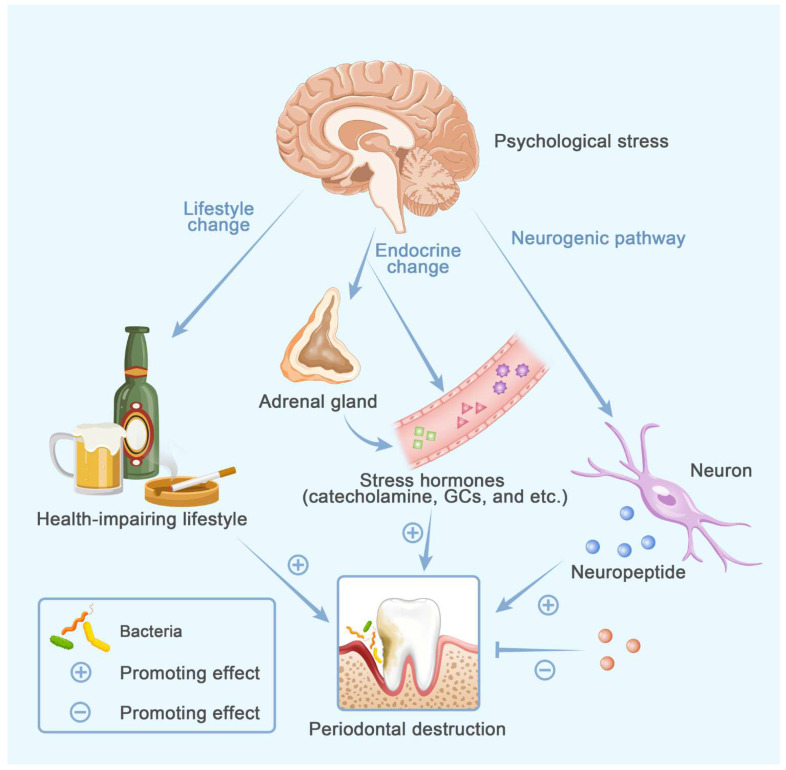
Impacts of psychological stress on periodontal status. Psychological stress affects periodontitis via three pathways, including lifestyle change, endocrine change and neurogenic pathway. Health-impairing lifestyles, such as smoking and alcohol consumption, exacerbate periodontitis-associated alveolar bone loss. Endocrine change mainly manifests as altered levels of stress hormones, such as GCs and catecholamine. GCs and catecholamine modulate bone remodeling and immuno-inflammatory response, adding to periodontal deterioration. Neuropeptides are the major effectors in the neurogenic pathway. Current evidence indicates the presence of both protective and harmful factors among neuropeptides. GCs, glucocorticoids.

Psychological stress mostly results from adverse events which exceed individual coping ability, causing corresponding reactions from both physiological and emotional perspectives [320]. Chronic psychological stress brings about complex pathophysiologic and behavioral changes. Stress modulates the activity of SNS and induces abnormal alternation of multiple endocrine hormones, such as GCs, PTH and gonadal hormones. Under stressful situations, people tend to acquire health-impairing habits, including abuse of cigarettes, alcohol and junk food [321]. Psychological stress not only has been indicated as a risk factor for osteoporosis by a bunch of evidence [321], but also positively associates with both the presence and severity of periodontitis [78,322]. Some inconsistent findings on the relationship between periodontitis and stress might ascribe to the failure to adapt potent parameters in stress assessment, such as the evaluation of invalidated subjective parameters and salivary cortisol which often fluctuates in a wide range [78]. It is plausible that the synergetic effects of those pathophysiologic and behavioral reactions mentioned above modify immuno-inflammatory response and periodontal plaque biofilm, adding to vulnerability to osteoporosis and periodontitis. A majority of these contributing factors have been elucidated in the previous charts. Herein, we are dedicated to finding out how the rest of the components, especially neurogenic factors, are involved in the pathologies of osteoporosis and periodontitis (Figure 5) (Table 4).

### Neurogenic Factors in Osteoporosis and Periodontitis

Considerable innervation density of sensory and sympathetic nerve fibers is observed in bone, primarily in the periosteum, bone marrow, and sites that are closest to bone surfaces that exhibit high bone turnover. Periodontal bone and soft tissues are also densely innervated by peptidergic nerve fibers, including sensory nerves and sympathetic nerves [335]. The sensory nervous system is tightly related to osseous pain. SNS is one of the pivotal downstream pathways that are activated by physiological stress besides the HPA axis. In response to sustained stressors, hyperactive SNS results in the secretion of catecholamines (adrenalin/noradrenalin), chromogranin A and neuropeptides, including substance P (SP), neuropeptide Y, vasoactive intestinal peptide (VIP), neuromedin and, etc. These SNS-derived molecules have been recognized as stress markers. Additionally, they have demonstrated the capability of modulating bone homeostasis, which may be the potential mechanisms underlying psychological stress-associated bone loss [323]. Emerging evidence supports that periodontitis is correlated with the homeostatic imbalance of neuropeptides. Neuropeptides in GCF are observed to be altered by periodontal status. Periodontitis-affected sites present elevated SP and VIP and decreased calcitonin gene-related peptide (CGRP) in GCF compared with those in healthy controls [335]. Denervation via inferior alveolar nerve transection modulated expressions of neuropeptides, CGRP and SP, subsequently aggravating periodontal bone loss and slowing the regeneration process [324].

Catecholamines and most of those neuropeptides inhibit bone formation and promote bone resorption via specific receptors on osteoblasts and osteoclasts, therefore damaging bone mass and bone microstructure [321,325,326]. However, VIP exhibits bone-sparing properties. The role of neuropeptide Y in bone biology is confusing. Aside from direct suppression of osteoblast differentiation, it is also found to eliminate mental stress-related bone loss [323]. Periodontal CGRP is secreted by sensory neurons with great potency in inhibiting alveolar bone resorption and promoting bone regeneration. SP and periodontitis could evoke enzyme-mediated degeneration of CGRP, thus blunting the protective effects of CGRP on periodontal bone [335].

It is suggested that besides bone remodeling, the immune defense system is also regulated by activated peptidergic neurons. VIP and SP display opposing effects on regulating the immune response. VIP inhibits inflammation response and decreases the local RANKL/OPG ratio, therefore mitigating periodontal bone loss induced by *Escherichia coli* in rats [327]. Conversely, SP is verified to be a pivotal mediator in periodontitis exacerbation under conditions of mental stress via altering inflammatory cytokines production, upregulating hypoxia-inducible factor 1 alpha and increasing the RANKL/OPG ratio [328,335]. Furthermore, chromogranin A is shown to possess antibacterial properties. SNS activation stimulates the secretion of alpha-amylase, a saliva enzyme, which reinforces the oral mucosa barrier against bacterial invasion [325].

Of note, the impact of catecholamines on periodontitis should be specified. SNS-derived catecholamines activate adrenergic signaling [329]. The β adrenergic signaling predominantly mediates the direct catabolic actions of activated sympathetic nerves on bone and contributes to periodontal bone destruction in the context of periodontitis [323]. In a mice model with apical periodontitis and chronic psychological stress, inhibition of sympathetic signaling with either α- or β-adrenergic blockers resulted in declined numbers of osteoclasts in the periapical lesion. However, inflammatory cytokines did not show a significant difference compared to controls [330]. Similar phenomena were also observed in rats with periodontitis and adrenergic signaling blockade, in which β-adrenergic blockers seemed not to affect the inflammation profile but to decrease well-differentiated osteoclast and consequent alveolar bone loss. Aside from that, activating β-adrenergic receptors ended up in reduced lingual alveolar bone [331,332]. Whereas chemical sympathectomy with neurotoxic drug 6-hydroxydopamine, which damages noradrenaline nerve terminals to restrain the release of noradrenaline (a neurotransmitter), modified immune response and mitigated alveolar bone loss induced by ligature [333]. Altogether, the adrenergic signaling pathway indeed promotes osteoclastic alveolar bone resorption. However, there are paradoxical findings in terms of the immunoregulatory effect of the adrenergic signaling pathway. Different methods adopted to block the adrenergic signaling among these experiments, including antagonist administration and chemical sympathectomy, partly account for the inconsistency. Since the chemical sympathectomy method is more complete in blocking adrenergic signaling, the viewpoint that the adrenergic pathway actually modifies immune response seems to be more convincing. Like other mental stress-related hormones, catecholamine hormones also benefit the growth of specific subgingival bacteria by stimulating autoinducer production or depleting exogenous iron in the periodontal microenvironment [334].

In this review, we try to provide all available evidence to date regarding how risk factors modify osteoporosis and periodontitis. Nevertheless, the effects of certain factors are relatively poorly studied, and a systematic review of current evidence is lacking. Moreover, psychological stress and GSs in hormones are closely correlated, independent analysis of each factor might be difficult. In the future, more studies are needed to better clarify the mechanistic link between osteoporosis and periodontal bone health, with more comprehensive and logical classification for their modifying factors.

## 6. Conclusions

Osteoporosis and periodontitis are both chronic diseases with high prevalence rates, manifested by the deterioration of systemic bone tissues or periodontal connective and bone tissues. Although available evidence indicates that the presence of osteoporosis correlates with periodontitis risk, an overview of the roles of predisposing factors in osteoporotic individuals in the pathological process underlying periodontitis is still lacking. This paper unveils the links between periodontitis and osteoporosis from the perspective of shared risk factors. These risk factors include hormones (sex hormones, calciotropic hormones, circadian rhythm-associated hormones, GH and TH), metabolic disorders (glucose metabolism, lipid metabolism and AAs metabolism), unhealthy lifestyle (smoking and excessive alcohol consumption) and psychological stress. The other thing is that we must keep in mind that the actions of those predisposing factors are not independent but intricate interactions and cross-talks. Future studies are required to provide deeper insight and close the knowledge gap in our current understanding of the respective and interactive actions of those factors on bone and periodontal health.

## Figures and Tables

**Table 1:** Impact of hormones on osteoporosis and periodontitis.

Hormone			Osteoporosis	Periodontitis			
				Overall	Bone Cells	Resident Cells (PDL Cells)	Others
	**Androgen** [1,14,15,16,17,18,19,20,21,22,23,24,25]	Normal	Normal level↓ Others unclear	↓	OBs↑ Osteocytes↑	Unclear	Pro-inflammatory cytokines (IL-1β, IL-6)↓
		High		↑	OCs↑	Unclear	Biofilm pathogenity↑ Pro-inflammatory cytokines (IL-1β)↑
	**Estrogen** [26,27,28,29,30,31]	Sufficient	↓	↓	OBs↑ OCs↑	Osteoblastogenesis↑	Pro-inflammatory cytokines (TNF-α, IL-1, IL-6, RANKL, PGE2, IL-8)↓
		Deficient	↑	↑	OCs↑	Unclear	Pro-inflammatory cytokines (IL-33, TNF-α, IL-1β)↑
	**Progesterone** [18,32,33,34,35]		↓	Unclear	OBs↑ OCs unclear	Osteoblastogenesis↑	*P. gingivalis*↑ Pro-inflammatory cytokines (IL-6↓, PGE2↑)
	**FSH** [11,36,37,38,39]		↑	↑	OCs↑ OBs unclear	Unclear	Pro-inflammatory cytokines (IL-1β, IL-6, TNF-α)↑
	**Vitamin D** [40,41,42,43,44,45,46,47,48,49,50,51,52,53]		↓	↓	OBs↑ OCs unclear	Mineralization activity↑	Bacterial loads↓ Degeneration-associated factor (MMP)↓ Cytotoxic T lymphocytes↓ Pro-inflammatory cytokines (IL-4, IL-6, IL-8, IL-10, TNF-α, MCP-1)↓ Anti-microbial properties↑ Anti-inflammatory cytokine (IL-17)↑
	**PTH**	Intermittent PTH [54,55,56,57,58,59,60,61,62,63,64]	↓	↓	OBs↑ OCs↑ Osteocytes↓	Immature PDL cells: cell number↓ osteogenesis↑	Degeneration-associated factors (IL-6, MMP-2, MMP-9)↓
						Mature PDL cells: cell number↑ osteogenesis↓	
		Continuous PTH [54,57,65,66]	↑	Unclear	OCs↑ Osteocytes↓	Osteoblastogenesis↑ (transient)	Pro-inflammatory cytokines (PGE2 transiently↓, then↑)
	**Calcitonin** [67,68,69,70,71,72,73,74]		↓	↓	OBs↓ OCs↓	Osteoblastogenesis and secretion activity↑	Bone remodeling coupling↑ Pro-inflammatory cytokines (PGE2)↓
	**GCs** [75,76,77,78,79,80]		↑	↑	OBs↓ OCs↑ (transient)	Unclear	Immunosuppressive property: Th1 response↓ Th2 response↑ Anti-inflammatory property: LPS signaling↓ Receptor for GCs↓
	**Melatonin** [81,82,83,84,85,86,87,88,89,90,91,92,93]		↓	↓	OBs↑ OCs↓	Unclear	Pathogenicity and formation of biofilm↓ Pro-inflammatory cytokines↓ Th1 cells migration↓ Oxidative stress↓ Angiogenesis↑ Glucose homeostasis and circadian rhythms↑
**TH**	Hyperthyroidism [94,95,96,97,98,99,100,101,102,103]		↑	↑	OBs↑ OCs↑	Unclear	Periodontal flora imbalance↑ Oxidative stress and ROS↑ Neutrophil apoptosis↑ Inflammatory cytokines (IL-6, TNF-α)↑
	Hypothyroidism [94,98,99,100,101,102,103,104,105,106]		Unclear	↑			
**GH**	Excess [107,108,109,110,111,112,113,114]		↑	↓	OBs↑ OCs↑	Osteogenic potential↑	Inflammatory response and collage metabolism (IL-1, IL-10, carboxyterminal telopeptide of type I)↓
	Deficiency [108,109,110,111,112,113,114,115,116]		↑	↑			Inflammatory mediators (CRP, MMP-8 and IL-8)↑

↑, Disease/cellular activity/molecular level is enhanced; ↓, Disease/cellular activity/molecular level is inhibited; Unclear, lack of conclusive evidence. OBs, osteoblasts; OCs, osteoclasts.

**Table 2:** Impact of metabolic disorders of energy substrates on osteoporosis and periodontitis.

Metabolic Disorders of Energy Substrates		Osteoporosis	Periodontitis			
			Overall	Bone Cells	Resident Cells	Others
**Glucose** **(hyperglycemia)** [151,152,153,154,155,156,157,158,159,160,161,162,163,164,165,166,167,168,169,170,171,172,173,174,175,176,177]		↑	↑	OBs↓ OCs Unclear Osteocytes↓	Osteogenic differentiation and proliferation of PDL stem cells↓	Energy fueling Collagen brittleness↑ Local pathogen loads↑ immune-inflammatory response: (1) Immune cells: Inflammation infiltration↑ Senescence, pyroptosis, immunocompromise and M1 polarization of macrophages↑ (2) Cytokines: Pro-inflammatory cytokines (TNF-α, IL-1β, IL-6, IL-4, IL-10, IL-17)↑ Anti-inflammatory cytokines (IL-10, FGF-21, MCP-1, TNF-β)↑
**Lipid**	**Hyperlipidemia** [178,179,180,181,182,183,184,185,186,187,188,189,190,191,192,193,194,195,196,197,198,199,200,201,202,203,204,205,206,207]	↑	↑	OBs↓ OCs↑	Unclear	Alternative energy source Pathogen loads↑ Inflammatory cytokines and cells↑ Lipid oxidation and oxidative damage↑
	**High SFA/UFA ratio** [178,183,191,205,208,209,210,211,212,213,214,215,216,217,218,219]				Survival and osteogenic differentiation of PDL fibroblasts↓	Inflammatory cytokines↑ Local immune cells (Neutrophil, monocyte, lymphocyte)↑
**AAs**	**BCAAs,** **aromatic AAs** [220,221,222,223,224,225,226,227,228,229,230,231,232,233,234,235,236]	↓	Unclear	OBs↑	Unclear	Alternative energy source for bone cells Prime energy substrates for some bacteria
	**Sulphur-containing AAs** [221]	↑		OBs↓ OCs↑		

↑, Disease/cellular activity/molecular level is enhanced; ↓, Disease/cellular activity/molecular level is inhibited; Unclear, lack of conclusive evidence. OBs, osteoblasts; OCs, osteoclasts.

**Table 3:** Impact of lifestyle on osteoporosis and periodontitis.

Lifestyle		Osteoporosis	Periodontitis			
			Overall	Bone Cells	Resident Cells	Others
**Smoking** [261,262,263,264,265,266,267,268,269,270,271,272,273,274,275,276,277,278,279,280,281,282,283,284,285,286,287]		↑	↑	OBs↓ OCs↑	Viability and osteogenic potential of MSCs and PDL cells↓	Subgingival microbial dysbiosis↑ Innate and adaptive host immune defense: compromised Salivary antimicrobial peptides↓
**Alcohol** **Consumption** [288,289,290,291,292,293,294,295,296,297,298]	**Low/** **moderate**	Unclear	↓	Unclear	Unclear	Unclear
	**Heavy**	↑	↑	OBs↓ OCs↑		Subgingival microbial dysbiosis↑ Disturbed redox homeostasis

↑, Disease/cellular activity/molecular level is enhanced; ↓, Disease/cellular activity/molecular level is inhibited; Unclear, lack of conclusive evidence. OBs, osteoblasts; OCs, osteoclasts.

**Table 4:** Impact of psychological stress on osteoporosis and periodontitis.

Psychological Stress		Osteoporosis	Periodontitis			
			Overall	Bone Cells	Resident Cells	Others
**Behavioral factors**		↑	As shown in Table 3			
**Physiological factors**	**Endocrine factors**		As shown in Table 1			
	**Neurogenic** **factors** [322,323,324,325,326,327,328,329,330,331,332,333,334]		↑	OBs↓ OCs↑	Unclear	Modify immuno-inflammatory response Dysbacteriosis↑

↑, Disease/cellular activity/molecular level is enhanced; ↓, Disease/cellular activity/molecular level is inhibited; Unclear, lack of conclusive evidence. OBs, osteoblasts; OCs, osteoclasts.

## Data Availability

Data sets used and/or analyzed during the current study are available from the corresponding author on reasonable request.
